# Toward robust and reproducible pluripotent stem cell expansion in bioprinted GelMA constructs

**DOI:** 10.36922/ijb.4633

**Published:** 2024-12-10

**Authors:** Elizabeth R. Komosa, Wei-Han Lin, Brenda M. Ogle

**Affiliations:** 1 Department of Biomedical Engineering, College of Science and Engineering, University of Minnesota, Minneapolis, Minnesota, United States of America; 2 Stem Cell Institute, University of Minnesota, Minneapolis, Minnesota, United States of America; 3 Lillehei Heart Institute, University of Minnesota, Minneapolis, Minnesota, United States of America; 4 Department of Pediatrics, Medical School, University of Minnesota, Minneapolis, Minnesota, United States of America; 5 Institute for Engineering in Medicine, University of Minnesota, Minneapolis, Minnesota, United States of America; 6 Masonic Cancer Center, University of Minnesota, Minneapolis, MN, United States of America

**Keywords:** Bioprinting, Scaffold characterization, GelMA, Human induced pluripotent stem cells, *In situ* expansion

## Abstract

Combining the technologies of 3D bioprinting and human induced pluripotent stem cells (hiPSCs) has allowed for the creation of tissues with organ-level function in the lab, a promising technique for disease modeling and regenerative medicine. Expanding the stem cells in bioprinted tissues prior to differentiation allows for high cell density, which is important for the formation of cell-cell junctions necessary for macroscale function upon differentiation. Yet, stem cell expansion, critical to successful *in situ* differentiation, depends heavily on the composition of the bioprinted scaffold. Here, we demonstrate how a common bioink component, gelatin methacryloyl (GelMA), varies depending on the vendor and degree of functionalization. We found that the vendor/GelMA production technique played a greater role in dictating the mechanical properties of the bioprinted constructs than the degree of functionalization, emphasizing the importance of reporting detailed characterization of GelMA scaffolds. Furthermore, the ability of singularized hiPSCs to survive and expand in GelMA scaffolds greatly varied across batches from different vendors and degrees of functionalization, where expansion correlated with the mechanical properties of the scaffold. Yet, we found that using a commercial cloning supplement could restore the ability of single hiPSCs to survive and expand across GelMA types, thus compensating for the varied mechanical properties of the scaffolds. These findings provide a practical guide for the expansion of hiPSCs in GelMA constructs with various mechanical properties as required for successful *in situ* differentiation.

## 1. Introduction

The field of tissue engineering has rapidly grown with the advancement of both human induced pluripotent stem cells (hiPSCs) and three-dimensional (3D) bioprinting. The ability to create human-relevant disease models and to fabricate 3D tissues has opened the door for many applications, including basic biology studies of tissue- and organ-level function and disease mechanisms, as well as translational uses in drug screening and regenerative medicine. A recent field trend combines hiPSCs and 3D bioprinting, where stem cells are bioprinted,^1^ expanded,^2–6^ and then differentiated in the bioprinted tissue^7–12^—a process termed *in situ* expansion and differentiation (ISED). ISED in 3D tissues boasts many benefits, including enhanced biomimetic environments over two-dimensional (2D) culture and the formation of cell-cell and cell-matrix junctions, which are important for macroscale tissue function.^7,9–11,13–17^ For instance, in bioprinted tissues, ISED enabled the fabrication of a 3D human cardiac chamber model capable of pump function.^10^ A pump of this type allowed for first-time measurements of clinically relevant pressure-volume relationships and the inclusion of healthy and diseased human cell types. Similarly, the neural application of ISED led to spontaneous action potential recordings that were not seen in 2D cultures, as well as metabolic switching that demonstrated early and advanced maturation in the 3D cultures.^17^ Importantly, robust and reproducible implementation of the ISED approach is dependent on the properties of the scaffold and the health of the printed stem cell population.

Gelatin methacryloyl (GelMA) is one of the most common base components of bioinks used in scaffold fabrication and bioprinting, including the ISED approach,^6,10–12,14,16^ as its natural and synthetic components allow for tunable properties, cell binding motifs, and printability.^18^ Tunable properties include matrix stiffness and pore size, altered by the source of raw gelatin, degree of gelatin functionalization (DoF), and GelMA concentration in the bioink. Further flexibility of GelMA bioinks has been demonstrated via supplementation with other potent factors for controlling stem cell activity.^10,14^ However, the high batch-to-batch variability of GelMA makes reproducing results challenging and reduces the translatability of studies. Fabrication protocols vary among groups producing GelMA in-house and commercially. Even when the same fabrication conditions are utilized across groups, large variability in scaffold properties— may still happen—in a systematic review, DoF ranging from ~70% to ~99% was observed when the same approach was used by four different groups.^19^ Scaffold properties (*e.g.*, DoF, swelling, and storage modulus) can diverge depending on synthesis conditions, including temperature, rate and amount of methacrylic anhydride addition, and stirring speed.^19^ Further, several commercial products do not specify DoF, and vendors that do characterize DoF often use different testing parameters and methodologies, making it difficult to predict behavior for desired applications.

The use of human pluripotent stem cells (hPSCs) adds further complexity, as their pluripotent nature makes them particularly sensitive to local environments. For instance, hPSCs are highly susceptible to dissociation-induced cell death due to myosin hyperactivation, triggered by activation of Rho signaling upon singularization.^20,21^ However, singularization of cells is common in extrusion bioprinting, currently the most common bioprinting modality, making hiPSCs difficult to employ in this context.. Inhibition of Rho-associated kinases (ROCK), via molecules such as Y-27632 and Fasudil, have helped to reduce cell death with singularization,^22^ though high viability remains a challenge in 3D cultures. Moreover, substrate mechanics also impact both hPSC proliferation and differentiation.^23,24^ For instance, Labouesse *et al.*^25^ examined the impact of substrate stiffness on pluripotent stem cell gene expression, where principal component analysis on differentially expressed genes led to samples clustering by substrate stiffness rather than the density of tethered extracellular matrix proteins. While investigating the effect of substrate stiffness and mechanotransduction during early mesoderm induction Smith *et al.*^26^ found that stiff substrates reduce β-catenin expression, a key component in many differentiation pathways, in early mesoderm induction, and discovered improved endothelial specification on compliant surfaces. These results demonstrate that the mechanical properties of a scaffold are likely to impact ISED outcomes.

Given the variability in GelMA characteristics and the impact of the microenvironment on stem cell culture, it is not surprising that inconsistencies in hiPSC expansion arise when switching between vendors and lots of GelMA. Variable expansion is a barrier to successful ISED, making it difficult to achieve consistent differentiation outcomes and therefore consistent tissue function. Here, we investigate two primary parameters that dictate the successful implementation of the ISED approach, namely GelMA material properties and hiPSC expansion capabilities. In this paper, we share some accessible means to monitor material properties and hiPSC expansion. Through the present study, we found that some GelMA products allow for improved hiPSC survival and expansion over others, a change in construct storage moduli accompanies cell expansion, and the addition of a cloning supplement post-print allows hiPSCs to survive and proliferate over a wider range of GelMA properties. These results will aid in future studies utilizing ISED for a range of applications.

## 2. Methods

### 2.1. Bioink preparation and bioprinting

Acellular bioink was made by dissolving 5, 7.5, or 10% w/v GelMA and 0.5% w/v lithium phenyl-2,4,6-trimethylbenzoylphosphinate (LAP; Allevi, USA) in a solution of 75% v/v mTeSR1 (cat#85850, STEMCELL Technologies, Canada) and 25% v/v acetic acid (20 mM) at 60°C. For cellular constructs, precursor ink was made with 10 or 20% w/v GelMA and 1% w/v LAP, which was mixed 1:1 with hiPSCs for a final concentration of 15 million cells/mL in 5% or 10% GelMA, respectively, and 0.5% LAP. Ink sources and lots are listed in Table 1. Three GelMA types were sourced from either a commercial supplier (“C”) or an in-house facility (“H”), with two different DoFs (low 50% vs high >90% ). Each GelMA type included 2–3 lots (“1,” “2,” or “3”) for comparison. All GelMA products were derived from 300-bloom type A porcine gelatin to maintain consistency in the gelatin source, with only the production method and DoF varying across samples.

G-codes for the bioprinted constructs (10 × 10 mm^2^, 1.2 mm thick) were generated in Slic3r, with 100% rectilinear infill. Multiple constructs were printed per g-code; constructs were deposited in a 30 mm dish containing a support bath. Support bath (FRESH v2.0)^27^ was created using gum arabic and gelatin type B, as previously reported.^6^ The diameters of support bath particles were quantified in ImageJ across 4 batches, 30 particles per batch. Bioprinting was done using an INKREDIBLE+ bioprinter (CELLINK, Sweden). To account for changes in viscosity among ink sources, the drop rate of ink from the needle was used to set the pressure,^10^ where 3.1 ± 0.3 s between drops was found to give construct structures representative of the g-code. Viscous inks (10% inks from Advanced Biomatrix, USA) were printed at 32°C, and less viscous inks (5% inks from Advanced Biomatrix, USA; and _10%_ inks from Bioprinting Facility, University of Minnesota) were printed at 27°C. Upon completion of the g-code, the constructs were cross-linked with 405 nm blue light for 20 s each above and below the construct. The support bath was then melted by incubating at 37°C for 30 minutes. Constructs were rinsed three times with warm Dulbecco’s PBS (DPBS; no calcium or magnesium, cat#14190144, Thermo Fisher Scientific, USA). Cast samples were made by pipetting 100 μL of 10% C-hi1 ink in circular 8 mm-diameter polydimethylsiloxane (PDMS) molds.

### 2.2. Swelling behavior

To assess the swelling behavior of the bioprinted constructs, the masses of acellular constructs were measured after 1 day of culture at 37°C in either mTeSR1 supplemented with 100 U/mL penicillin, 100 μg/mL streptomycin (pen-strep; Thermo Fisher Scientific, cat#15140122) or DPBS. Samples were then lyophilized in parafilm-lined dishes to prevent the wet gel from freezing into the dish. The mass swelling ratio (MSR) was calculated as the ratio of the wet mass (*Mw*) to the dry mass (*Md*) as expressed in Equation I:

(I) $$MSR=\frac{Mw}{Md}$$

Per GelMA type, 2–4 ink batches were tested, with 2–3 constructs per batch.

### 2.3. Mechanical characterization

The storage and loss moduli of the constructs were measured on a Discovery HR-3 rheometer (TA Instruments) with 8 mm parallel plates. Acellular constructs were tested on the day of bioprinting; while cellular constructs were evaluated on days 0, 7, and 14 after bioprinting. Adhesive P60 grit sandpaper (cat# DUR 9D-VP-10, DuraGold, TCP Global, USA ) was placed on the plates to minimize slippage of the constructs during testing. Biopsy punches (Acu-Punch, cat#0413, Acuderm, USA) were used to cut constructs and sandpaper into 8 mm circles. Frequency sweeps were performed at 1% strain and 37°C, ranging from 0.1 to 2.5 rad/s. The reported values were measured at 0.1 rad/s. For acellular constructs, 2–4 ink batches per GelMA type were tested, with 3–4 constructs per batch. For cellular constructs, 1–2 constructs were tested for each replicate, with 3 ink batches used.

### 2.4. Cell culture

hiPSCs overexpressing cyclin D2 under the myosin heavy chain gene^28^ (derived from a female donor; kindly provided by Dr Jianyi Zhang’s lab at the University of Alabama at Birmingham were maintained in mTeSR1 media on Matrigel (cat#08–774-552, Corning, United States) and passaged with ReLeSR (cat#05872, STEMCELL Technologies, Canada). For cell singularization for bioprinting, hiPSCs were incubated with Accutase (cat#A6964, Millipore Sigma, Germany), rinsed twice with mTeSR1, and resuspended at 30 million cells/mL in mTeSR1 with 10 μM Y-27632 ROCK inhibitor (cat#S1049, Selleckchem, United States). Cells were combined with ink as described above. Bioprinted constructs were incubated in mTeSR1 with 5 μM Y-27632 and pen-strep for the first day of culture, followed by mTeSR1 only, and changed daily for the remainder of culture.

### 2.5. Monitoring glucose consumption in hiPSC cultures and expansion

Glucose levels of the media were measured every 24 hours (± 30 minutes) for 14 days. A drop of the media sample was placed on parafilm, combining media from the same conditions across replicates, and glucose levels were recorded using a Contour Next One Blood Glucose Monitoring System (Ascensia Diabetes Care, Switzerland). Glucose consumption was approximated by subtracting the measured glucose levels from the basal glucose levels of the media, which were recorded on day (d) 1 of the experiment or upon using a newly supplemented bottle of mTeSR1.

### 2.6. Histological preparation and immunostaining of constructs for cellular analysis

At the end of the 14-day culture period, constructs were fixed overnight at 4°C in 4% paraformaldehyde. Cells were prepared for cryosectioning as previously described.^6^ Sections were stained with 5 μg/mL 4′,6-diamidino-2-phenylindole (DAPI; cat#D1306, Invitrogen, United States) for 10 minutes and mounted with a solution of DAPI/1,4diazabicyclo[2,2,2]octane (DABCO; cat#D27802, Sigma-Aldrich, United States) in glycerol and PBS. Tile scan imaging was performed on a Leica Dmi8 fluorescence microscope, and DAPI colonies ≥ 2500 μm^2^ were quantified and normalized to tissue area, as determined by brightfield images. Eight sections per construct were analyzed; four from the first 0–500 μm of the tissue and four from the 500–1000 μm of tissue depth, with 1–3 constructs per condition per print batch (*n* = 3).

For immunostaining, one construct per condition per print batch was stained for OCT3/4 and SP1 expression. Samples were permeabilized with 0.2% Triton X-100 (cat# T8787, Sigma-Aldrich, United States) for 1 hour and blocked with a solution of 5% bovine serum albumin, 1% glycine, 2% goat serum, and 0.1% Triton X-100 (BGST) for 2 hours. Mouse anti-OCT3/4 (1 μg/mL; cat#sc-5279, Santa Cruz Biotechnology, Country) and rabbit anti-SP1 (2.2 μg/mL; cat#PA5–29165, Invitrogen, United States) in BGST were incubated overnight at 4°C. Sections were then washed with 0.2% Tween-20 (cat#655204, Millipore, Germany) in PBS and incubated for 2 hours with secondary antibody (4 μg/mL AlexaFluor 488 goat anti-mouse [cat#A32723, Invitrogen, USA] and 4ug/mL—AlexaFluor 647 goat anti-rabbit [cat#A32733, Invitrogen, USA]). After washing with Tween-20, samples were stained with DAPI and mounted as described above. Tile scans were captured, and OCT3/4 expression was normalized to SP1 to determine the pluripotency of living cells. F-actin was visualized using a 30-minute incubation with ActinGreen 488 ReadyProbes Reagent (cat#R37110, Invitrogen, USA) on permeabilized slides.

### 2.7. Evaluation of hiPSC expansion using Y-27632 and CloneR2 treatments in bioprinted constructs

To determine potential improvements for hiPSC expansion in the bioprinted constructs, C-lo1 GelMA was used. Following bioprinting, constructs were first cultured in mTeSR1 supplemented with Y-27632 (5 μM) for 1 or 2 days or in mTeSR1 supplemented with CloneR2 (1:10; cat#100–0691, STEMCELL Technologies, Canada) for 2 days, and then the media was changed back to fresh mTeSR1 for the remainder of hiPSC expansion period. CloneR2-containing media was neutralized with 1 M NaOH to ensure consistent pH between the two media types. Following these treatments, constructs were cultured under either static or dynamic conditions. In the dynamic culture, constructs were rocked at 30 rpm on a Lab-Line 4630 3-D Rotator (Marshall Scientific, USA). Glucose levels were monitored daily, and constructs were fixed on day 7 post-print. The experiment was repeated using C-hi1 (5%) GelMA, with 1 day of Y-27632 treatment under static or dynamic conditions, as well as under dynamic conditions with CloneR2 treatment for 2 days.

### 2.8. Evaluation of hiPSC differentiation following Y-27632 and CloneR2 treatments in bioprinted constructs

The differentiation potential of CloneR2-treated samples was evaluated in C-lo1 constructs using established cardiac differentiation protocols.^10,29^ Briefly, bioprinted constructs were first treated with either CloneR2 for 2 days or Y-27632 for 1 day as described above, and then after the proliferation period (7 days for CloneR2-treated constructs and 14 days for Y-27632-treated controls), cardiac differentiation was initiated with 12 μM CHIR99021 (cat#SML1046, Sigma-Aldrich, USA) in RPMI (cat#11875–093, Gibco, USA) supplemented with 1× B27 without insulin (cat#A1895601, Gibco, USA). After 24 hours, this media was replenished with fresh RPMI/B27 without insulin. On day 3 of differentiation, half of the exhausted media was collected from each condition and combined with fresh RPMI/B27 without insulin and supplemented with 5 μM IWP2 (cat#3533, Tocris, United Kingdom). On day 5 of differentiation, media was replaced with RPMI supplemented with 1× B27 with insulin (cat#1504001, Gibco, USA). Media was changed again on day 7 of differentiation, and constructs were fixed on day 10. Constructs were stained whole-mount for cardiac troponin T (1 μg/mL; cat#MS295P1, Epredia, USA) on a rocker and imaged on a Nikon A1Rsi confocal microscope (Nikon, Japan). Nine z-stacks were taken from each construct, and maximum intensity projections were created. Each condition had 2–3 print batches, with 1–2 constructs per batch.

To verify differentiation ability in more complex tissues, human chambered muscle pump (hChaMP) models were bioprinted and differentiated as previously described,^10^ with some modifications as follows: Rocking was performed at 3 rpm, and differentiation was initiated after 9 days of expansion. Videos demonstrating contractile function were recorded on a ZEISS Axiocam 208 microscope camera (ZEISS, Germany) on day 43 of differentiation.

### 2.9. Statistical analysis

Sample group comparisons were analyzed in JMP Pro 16.0 (JMP, USA). Normality was assessed with Shapiro–Wilk tests, and groups with normal distributions were compared using Student’s *t*-tests or analysis of variance (ANOVA) tests and post-hoc Tukey–Kramer honestly significant difference (HSD) tests. For non-normal datasets, Mann–Whitney tests or Kruskal–Wallis tests with post-hoc Steel–Dwass tests were performed. For comparisons to a control value (modulus change with cell growth or study of culture conditions to improve hiPSC expansion), post-hoc analyses were performed with Dunnett’s test (parametric) or Steel’s test (nonparametric). P-values were calculated for a null hypothesis of unequal means (two-tailed) with a 95% confidence interval.

## 3. Results

### 3.1. Hydration medium and fabrication method impact construct material properties

We first evaluated the impact of hydration medium on the MSR of GelMA constructs. We found that constructs cultured in DPBS showed a significantly higher MSR compared to those cultured in mTeSR1 stem cell medium (Figure S1a, Supplementary File). This demonstrates the importance of performing studies in relevant media, as studies performed in DPBS may overestimate swelling and media uptake.

Next, we compared hydrogel material properties between cast constructs and extrusion-printed constructs in a support bath. Cast constructs exhibited a significant reduction in MSR, approximately half of what was observed in mTeSR1, and a more than 16-fold increase in storage modulus (G′) compared to printed constructs (Figure S1b, Supplementary File). Importantly, the variability in MSR and storage modulus was lower in cast samples compared to printed samples, both within single batches and across different ink batches (Figure S1a and S1b, Supplementary File).

To account for slight variations in bioink viscosities, we set the print pressure for pneumatic extrusion based on the drop rate of the ink from the needle. The variability observed in the printed constructs is likely attributed to differences in support bath properties or errors in setting pneumatic pressure, rather than issues with GelMA solubilization across batch. The microparticles of the support bath (12.98 ± 0.80 μm) and yield stress (75.6 ± 34.1 Pa) were consistent across batches (Figure S2, Supplementary File), suggesting that these parameters were not significant contributors to variability in construct properties.

In contrast, we observed a wide range of set pressures required for printing among different ink batches (Figure S1c, Supplementary File), identifying pneumatic pressure as a primary source of variability in construct properties. This is supported by the clustering of bioprinted samples by ink batch in Figure S1a (Supplementary File), while pipetted samples showed no batch-specific separation. Therefore, pneumatic printing is identified as a key source of variability in construct properties.

### 3.2. Increasing GelMA concentration yields scaffolds with increased stiffness and decreased swelling

We evaluated the effect of GelMA concentration on MSR and storage modulus, two key parameters for tuning GelMA scaffolds. Constructs were tested at 5, 7.5, and 10% GelMA concentrations (C-hi1) and we observed that required print pressures decreased significantly with decreasing GelMA concentration, despite lower print temperatures for the 5% and 7.5% formulations (Figure S3a, Supplementary File). The constructs followed expected trends, with increasing GelMA concentration leading to reduced MSR and increased stiffness. Specifically, constructs with higher GelMA concentrations exhibited lower swelling and higher storage moduli (Figure S4, Supplementary File).

### 3.3. GelMA source and DoF significantly affect construct mechanical properties

**Figure 1.**
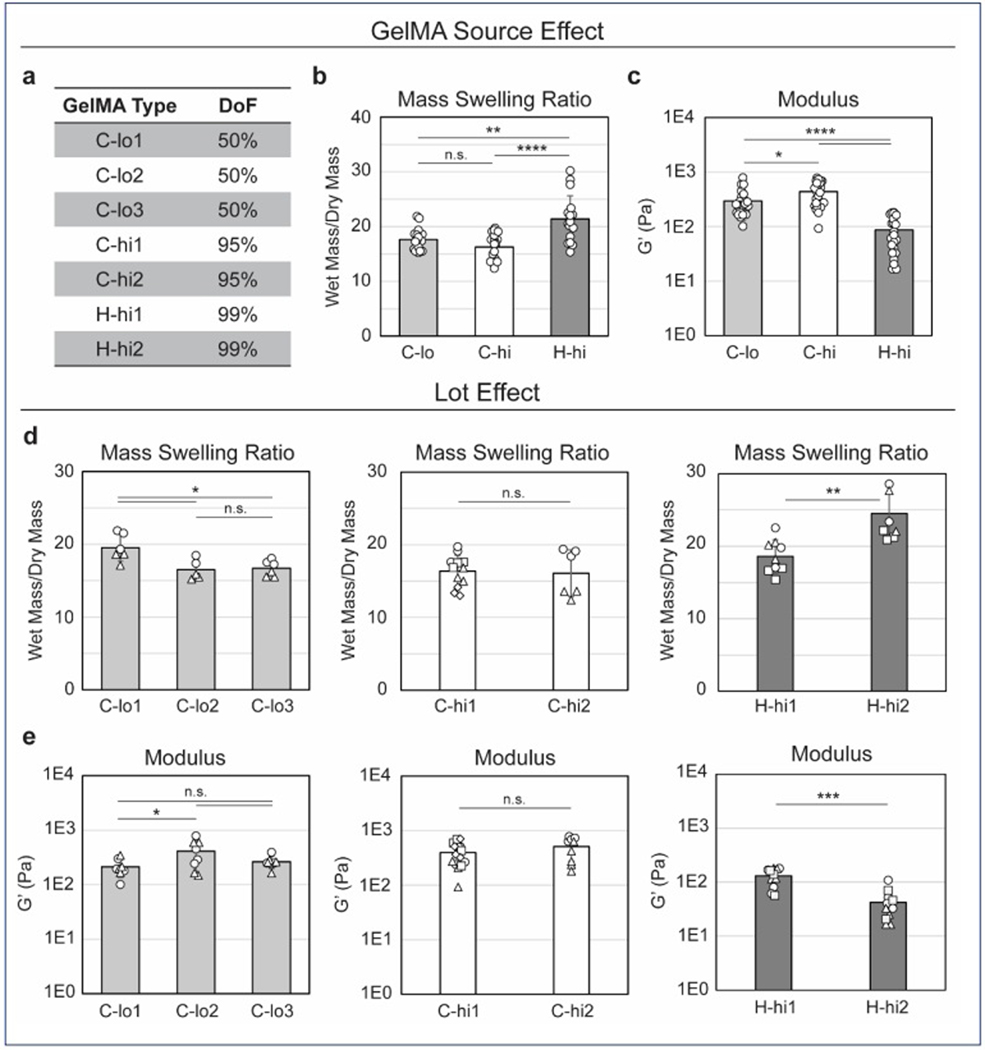
GelMA source and lot affect scaffold mass swelling and mechanical properties. (a) Summary of GelMA types. Labels are designated by source, DoF, and lot. Listed DoFs are obtained from product information. (b–c) Effect of GelMA type on MSR of acellular constructs after 1 day of culture in mTeSR1 (b) and G′ (c). Results show greater discrepancies between sources of GelMA than DoF. (d–e) Effect of GelMA lot on MSR (d) and G′ (e). Large variability was found between the two H-hi lots for both MSR and G′, and slight differences were seen among C-lo lots. (2–4 print batches per GelMA type, batches represented by the shape of data points on bar graphs, 2–3 constructs/batch for MSR, 3–4 constructs/batch for rheometry. *p ≤ 0.05, **p ≤ 0.01, ***p ≤ 0.001, ****p ≤ 0.0001, n.s. not significant.) Abbreviations: DOF: Degree of functionalization; GelMA: Gelatin methacryloyl; MSR: Mass swelling ratio.

We evaluated the impact of GelMA type and DOF on construct mechanical properties by evaluating three GelMA types (Table 1, Figure 1a). The GelMA types required a wide range of printing pressures (Figure S3b, Supplementary File), with source C inks needing over 90 kPa at 27°C. To standardize pressures, the print temperature for source C was raised to 32°C. The storage modulus (G′) and MSR were used to evaluate the mechanical properties of the GelMA constructs. A significant difference in MSR was observed between the C-hi and H-hi constructs, despite their similar DoF (Figure 1b). However, the MSR values of C-lo and C-hi constructs from source C were comparable. In terms of storage modulus, all three GelMA types demonstrated distinct values (Figure 1c). C-lo and C-hi constructs showed more similarity in G′ compared to the differences between C-hi and H-hi, indicating that the GelMA source and production method exerted a greater influence on construct stiffness than the DoF.

Interestingly, the differences in MSR and G′ among GelMA types were smaller than those caused by fabrication methods found in Figure S1a and S1b (Supplementary File). This suggests that the printing process plays a primary role in determining material properties of the construct, while the GelMA source is a secondary factor.

### 3.4. Lot-to-lot variability of GelMA depends on the manufacturing source

Breaking a given GelMA type down by a lot, we found some variability in MSR (Figure 1d) and G′ (Figure 1e). In general, the in-house-produced GelMA (H) exhibited greater lot-to-lot variability compared to the commercial products (C). No significant differences were found between the two C-hi lots, as MSR, G′, and set printing pressure were consistent (Figure S3c, Supplementary File). However, C-lo1 showed notable differences in MSR compared to C-lo2 and C-lo3, and in G′ compared to C-lo2. There was a non-significant increase in average printing pressure for C-lo2 relative to C-lo1 and C-lo3 (Figure S3c, Supplementary File). We also observed a large difference between MSR, stiffness, and print pressure H-hi1 vs. H-hi2, indicating significant lot variability that could affect downstream applications, such as hiPSC culture.

### 3.5. *In situ* expansion of hiPSCs is dictated by GelMA source and concentration

**Figure 2.**
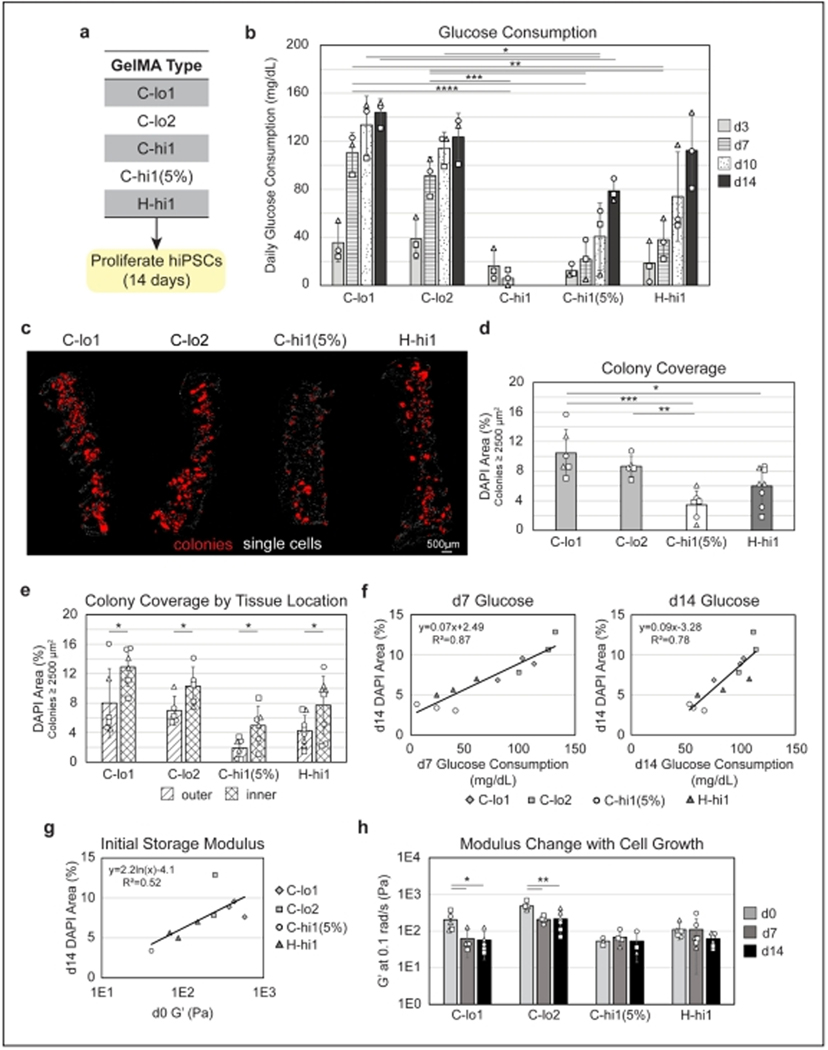
Selection of GelMA type impacts hiPSC expansion. (a) Summary of GelMA types tested for 14-day hiPSC expansion. (b) Glucose consumption of hiPSCs in each GelMA type increases over the 14-day period except C-hi1, which was terminated at d7. Most statistical significance between groups was found at d7 of proliferation. (c) Images of cross-sections at the end of the proliferation time, with nuclei stained with DAPI. Colonies, shown in red, are found to be the viable cells in the constructs, whereas single cells (white) remaining at d14 are found to be non-viable. (d) Quantification of colonies in d14 cross-sections shows the highest growth in C-lo1 and C-lo2 and the lowest in C-hi1 (5%), in accordance with the glucose consumption results. (e) Examination of colony coverage in outer (0–500 μm) vs. inner (500–1000 μm) portions of the constructs show higher cell growth in the interior of the constructs compared to the exterior. Yet, trends seen in panel (d) remain the same in both regions of the construct. (f) Glucose consumption from d7 (left) and d14 (right) show a strong correlation with the final colony coverage at d14, suggesting an accurate technique for non-invasively measuring hiPSC growth in 3D prior to initiation of differentiation. (g) A notable correlation also exists between the initial storage modulus at d0 of proliferation and the final colony coverage, regardless of the GelMA type used. (h) Change in G′ with cell growth. G′ changes more in C-lo inks, which are the inks with the most hiPSC expansion. (3 print batches per GelMA type, batches represented by the shape of data points on bar graphs, 1–3 constructs/batch. *p ≤ 0.05, **p ≤ 0.01, ***p ≤ 0.001, ****p ≤ 0.0001.) Abbreviations: GelMA: Gelatin methacryloyl; hiPSC: Human induced pluripotent stem cell.

Given the variability observed in GelMA sources and lots, we next aimed to determine how these differences might affect hiPSC viability and expansion. hiPSCs were cultured in different lots of C-lo GelMA (C-lo1 and C-lo2), two concentrations of C-hi1 (10% and 5%), and H-hi1, which exhibited similar storage modulus (G′) but different MSR compared to C-hi1 (5%) (Figure 2a). When printing with cells, a slight increase in print pressures was noted compared to the corresponding acellular inks (Figure S3d, Supplementary File). While this increase was non-significant for most GelMA types (as confirmed by *t*-tests comparing acellular and cellular inks), a two-way ANOVA showed that the presence of cells significantly influenced print pressure across different GelMA types and temperatures (*p* = 0.0026).

### 3.6. Monitoring glucose consumption for assessing hiPSC growth and expansion in GelMA constructs over 14 days

We successfully monitored the growth and expansion of hiPSCs in GelMA over a 14-day period through daily measurements of glucose levels (Figure S5, Supplementary File) in the exhausted media, with glucose consumption approximated by subtracting the consistent baseline readings of 272.0 ± 5.0 mg/dL from fresh mTeSR1 media. By evaluating the glucose consumption curves for each GelMA type (Figure S5a, Supplementary File), we saw a similar value on day 1 for all GelMA types, suggesting consistency in cell numbers among the initial ink compositions. Surprisingly, there was also a consistent drop between d1 and d2, likely due to the death of hiPSCs cultured as sparse, single cells. Cells began to recover at different rates, dependent on the GelMA type. Comparison of consumption values at d3, d7, d10, and d14 of culture revealed the greatest difference among the GelMA types occurring at d7, where the C-lo constructs differed from H-hi1 and C-hi1 at both concentrations (Figure 2b). Interestingly, hiPSCs did not expand well at 10% concentration for C-hi1, with glucose levels decreasing by d7 of proliferation, so the culture of these constructs was terminated at this time point and C-hi1 (5%) was used for subsequent cell studies. Overall, hiPSCs grew best in C-lo1 and C-lo2, with significantly improved glucose consumption at d5–d7 compared to C-hi1 (5%) and H-hi1, with lower variability in consumption rates at each timepoint, and fairly reproducible outcomes between the two GelMA lots. Glucose consumption of the C-lo inks tended to plateau around d10, suggesting this GelMA type may not require 14 days of proliferation prior to inducing differentiation with the ISED approach.

### 3.7. Differential colony coverage and localization in GelMA constructs after 14 days of hiPSC proliferation

Tissue sections obtained at d14 of proliferation were stained with DAPI to determine cell coverage. We found many singularized cells in all GelMA types (Figure 2c, *white* cells), and we suspected that since these cells had not formed colonies, they had likely died as a function of singularization in the printing process or due to lack of cell binding or nutrient access. Indeed, these singularized cells did not express F-actin or ubiquitous transcription factor SP1 (Figure S6, Supplementary File), confirming these cells had died early and remained trapped within the construct, similar to previous findings.^6^ Thus, only colonies (Figure 2c, *red* regions) were quantified for each tissue. We found 3-fold greater colony coverage in C-lo1 relative to C-hi1 (5%) and 1.7-fold more than H-hi1 (Figure 2d). When examining the two lots of C-lo GelMA, we found that C-lo2 had a ~12% decrease in the area of hiPSC colonies, though this difference was not statistically significant. Colonies remained pluripotent in all GelMA types (Figure S7, Supplementary File).

Interestingly, when comparing colonies in cryosections taken from outer tissue (~0–500 μm from construct surface) vs. inner tissue (~500–1000 μm from construct surface), there were more colonies in the inner than the outer tissue (Figure 2e). This is similar to previous findings when examining colony localization across a 3D-bioprinted chamber model and is attributable to cell detachment with support bath removal and media changes.^6^ Regardless, trends among colony coverage in the different GelMA types remained the same at both locations.

### 3.8. Correlation of glucose consumption and mechanical properties with hiPSC colony coverage in 3D-bioprinted GelMA constructs

We also examined the correlation between glucose consumption and final colony coverage, combining the values for all GelMA types to validate the utility of glucose consumption as an indicator of hiPSC expansion (Figure 2f; Figure S8, Supplementary File). The glucose consumption at d14 gave a high correlation to the colony coverage, as did consumption at d7 and d10. These data support the use of the glucose meter for easy quantification of cell growth in 3D. Using the earlier metrics to predict colony coverage at the end of the ISED proliferation period will be valuable to validating adequate expansion in a given GelMA type or lot and to standardizing the initiation of differentiation.

Another interesting trend we found by pooling all GelMA types was an increase in hiPSC colony coverage with increasing initial storage modulus (Figure 2g). As a relatively low range of G′ was used for these studies, we were surprised to see such a notable trend. This trend was also found with the initial loss modulus (G″; Figure S9a, Supplementary File). However, further study is needed to evaluate the effect of this phenomena, as there are several other variables relevant to the use of multiple GelMA types. For instance, there was a negative correlation between colony coverage and MSR, as well as between colony coverage and DoF (Figure S9b and S9c, Supplementary File). However, we did not see a trend between colony coverage and print pressure (Figure S9d, Supplementary File). Overall, the mechanical stiffness, affected by both DoF and MSR, appears to be a primary determinant of the viability and expansion of singularized hiPSCs.

### 3.9. Construct mechanical properties change with hiPSC expansion

During the 14-day hiPSC proliferation period, we monitored how cell growth impacted G′ and G″. Cellular constructs at d0 were comparable to the acellular constructs, showing the addition of cells to the ink did not initially affect construct properties. During *in situ* proliferation, the moduli of C-hi1(5%) and H-hi1 constructs did not change between d0 and d7, but both had slight, yet non-significant decreases between d7 and d14 (Figure 2h; Figure S10, Supplementary File). However, in C-lo1 and C-lo2 GelMA, we saw a drastic drop in both moduli by d7 of proliferation. Interestingly, there was no difference between the d7 and d14 moduli for these inks, during which time we found a less accelerated rate of glucose consumption compared to earlier days. Thus, these results correlate well with colony coverage and glucose consumption, suggesting that the cells reduce stiffness of the bulk tissue with volumetric expansion and likely cause some degree of remodeling. Examination of this impact is important for future studies of *in situ* differentiation, as substrate stiffness is an important regulator of cell morphology and can influence differentiation and maturation outcomes.

### 3.10. Use of CloneR2 improves hiPSC expansion in different GelMA types

**Figure 3.**
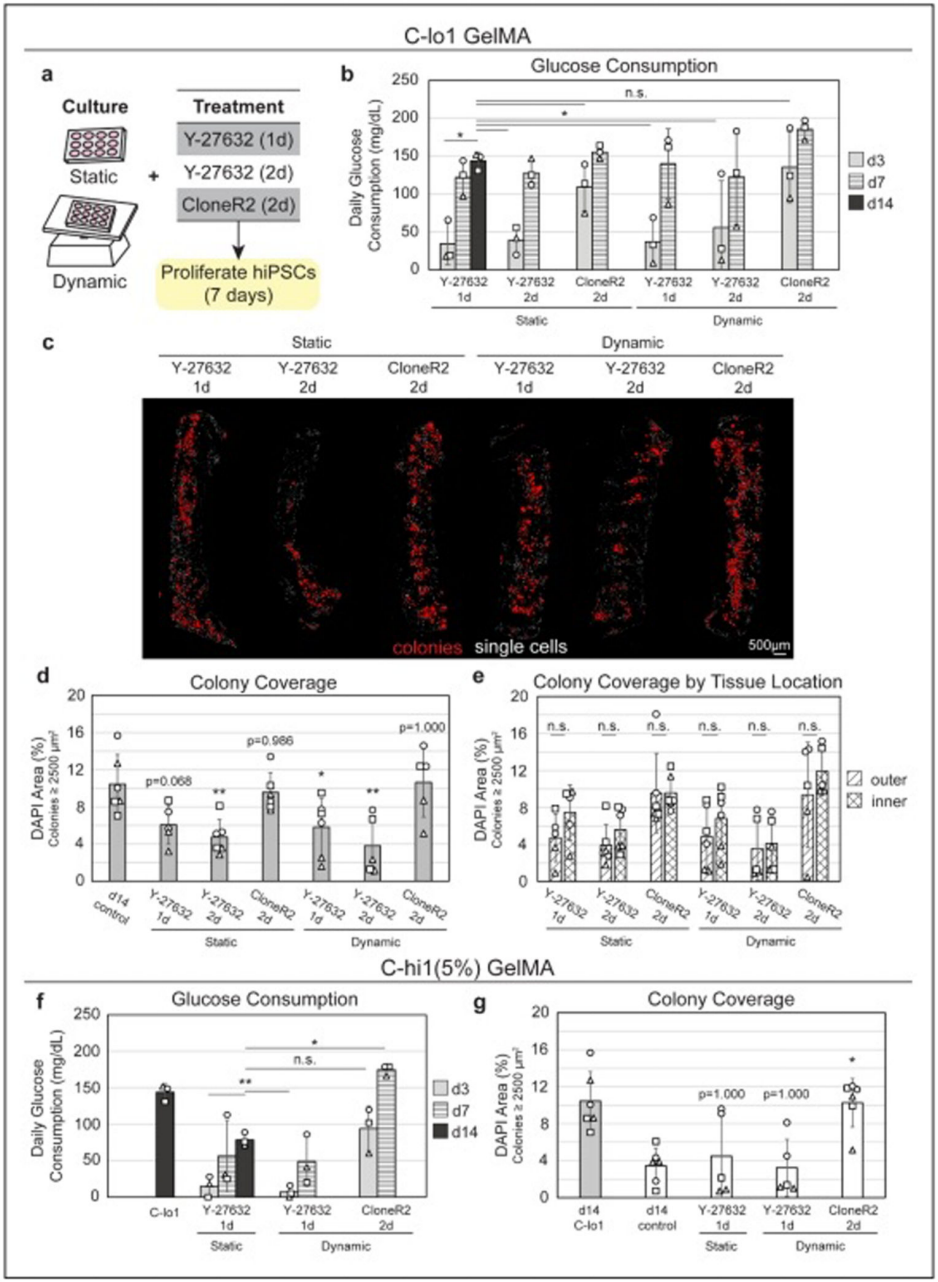
Early treatment with cloning supplement improves hiPSC expansion. (a) Summary of conditions tested with cells in C-lo1 GelMA. Cells were treated with Y-27632 for 1 or 2 days or with CloneR2 for 2 days, cultured for 7 days statically or dynamically on a rocker. (b) Glucose consumption of hiPSCs under each culture condition. d3 glucose consumption of CloneR2-treated samples do not differ from control samples at d14 (shown for reference). (c) Cross-sections showing colonies (red) after the 7-day proliferation, with nuclei stained with DAPI. Few single cells (white) remain in CloneR2-treated constructs. (d) Colony coverage after various culture condition treatments show similar growth in d7 CloneR2-treated samples relative to the d14 Y-27632-treated samples. (e) Colony coverage does not vary by region at d7 of culture in any culture condition. (f–g) Select conditions were tested with C-hi1 (5%) GelMA. Use of CloneR2 drastically improved hiPSC expansion, where d7 glucose measurements (f) and d7 colony coverage (g) far exceeded d14 C-hi1 on bar graphs, 1–2 constructs/batch. Significance was found relative to d14 samples of the same ink type using Dunnett’s test or Steel’s test. *p ≤ 0.05, **p ≤ 0.01, n.s. not significant.) Abbreviations: GelMA: Gelatin methacryloyl; hiPSC: Human induced pluripotent stem cell.

After observing a significant decline in glucose consumption at d2 of proliferation, we hypothesized that this early cell death could be reduced by altering our standard 1-day treatment with ROCK inhibitor, Y-27632, as used for the studies in section 3.5 to 3.9. A reduction in initial cell death may lead to better colony coverage at an earlier time point. Thus, we tested several culture conditions with C-lo1, the GelMA type that is the most supportive of hiPSC expansion. We tested both static and dynamic (rocking) culture, with 1- or 2-day treatment of Y-27632, as well as 2-day treatment with a defined stem cell cloning supplement, CloneR2 (Figure 3a; Figure S11a and S11b, Supplementary File). We again monitored glucose levels and still saw a drop at day 2 for all Y-27632 conditions in both static and dynamic cultures (Figure S11a, Supplementary File). However, with CloneR2-treated samples (under both static and dynamic culture), there was an increase in consumption at d2, and glucose consumption continued to increase, whereas the consumption in Y-27632 conditions remained relatively unchanged until ~d4–5. The CloneR2 conditions at d3 were not found to be significantly different from the d14 consumption values found for C-lo1 in Figure 2b. Furthermore, all replicates from the CloneR2-treated groups surpassed the d14 control average within 4 or 6 days of culture for dynamic and static conditions, respectively (Figure 3b), indicating a significantly accelerated expansion of hiPSCs with CloneR2 treatment. After 7 days of culture, constructs were fixed and sectioned for histology. Colonies were again found to reflect the glucose consumption profiles, where the CloneR2-treated samples at d7 held similar colony coverage to the Y-27632treated samples at d14 described in Figure 2d (Figure 3c–d). Fewer single (nonviable) cells appeared in CloneR2-treated samples, consistent with the increased glucose consumption on d2 of expansion. Relative to the d14 samples, CloneR2-treated samples had more colonies, which were generally smaller and more consistently sized, a possible advantage for differentiation outcomes.^48–50^ The addition of CloneR2 and the corresponding increase in growth rate did not affect the pluripotency of the hiPSCs (Figure S12, Supplementary File). Statistical comparisons between the colony coverage of d14 control (1d-treated Y-27632) and d7 CloneR2-treated samples yielded a p-value of > 0.98, indicating similar growth in half the proliferation time. Interestingly, despite the lower average in colony coverage of the d7 1d-treated Y-27632 constructs relative to the d14 timepoint, the two were not found to be significantly different (*p* = 0.068), possibly due to the large variability at each timepoint. The remaining treatment groups were significantly lower than the d14 control. Interestingly, unlike the 14-day studies in Figure 2, we did not see a significant difference between the inner and outer regions of the constructs with any d7 constructs (Figure 3e). This suggests that either colonies continue expanding more within the interior of the tissues than the exterior in the following 7 days or the colonies near the exterior detach. Regardless, this further demonstrates the utility of CloneR2-treated tissues, as we can obtain a similar colony coverage in half the culture time, with improved consistency between regions of the tissue. This improved colony distribution is important for engineering tissues for any application.

In further studies with the less proliferative C-hi1 (5%) GelMA, we found that dynamic Y-27632 culture did not alter glucose consumption or colony coverage relative to its static control (Figure 3f and g; Figure S11c and S11d, Supplementary File), suggesting the poor growth in C-hi1 (5%) is not due to poor nutrient supply. However, dynamic CloneR2 was again found to accelerate glucose consumption, where d3 glucose consumption was equivalent to the d14 control (1-day Y-27632 C-hi1 (5%) samples described in Figure 2) (Figure 3f; Figure S11c, Supplementary File). Surprisingly, d7 glucose consumption of CloneR2-treated constructs was more than double the amount of the d14 samples and even exceeded that found at d14 in the best GelMA type, C-lo1. Colony coverage reflected these trends, with CloneR2-treated constructs matching the d14 C-lo1 values and far exceeding the d14 C-hi1 (5%) values (Figure 3g).

### 3.11. Cardiomyocyte differentiation and functional cardiac tissue formation following CloneR2-supported hiPSC expansion

**Figure 4.**
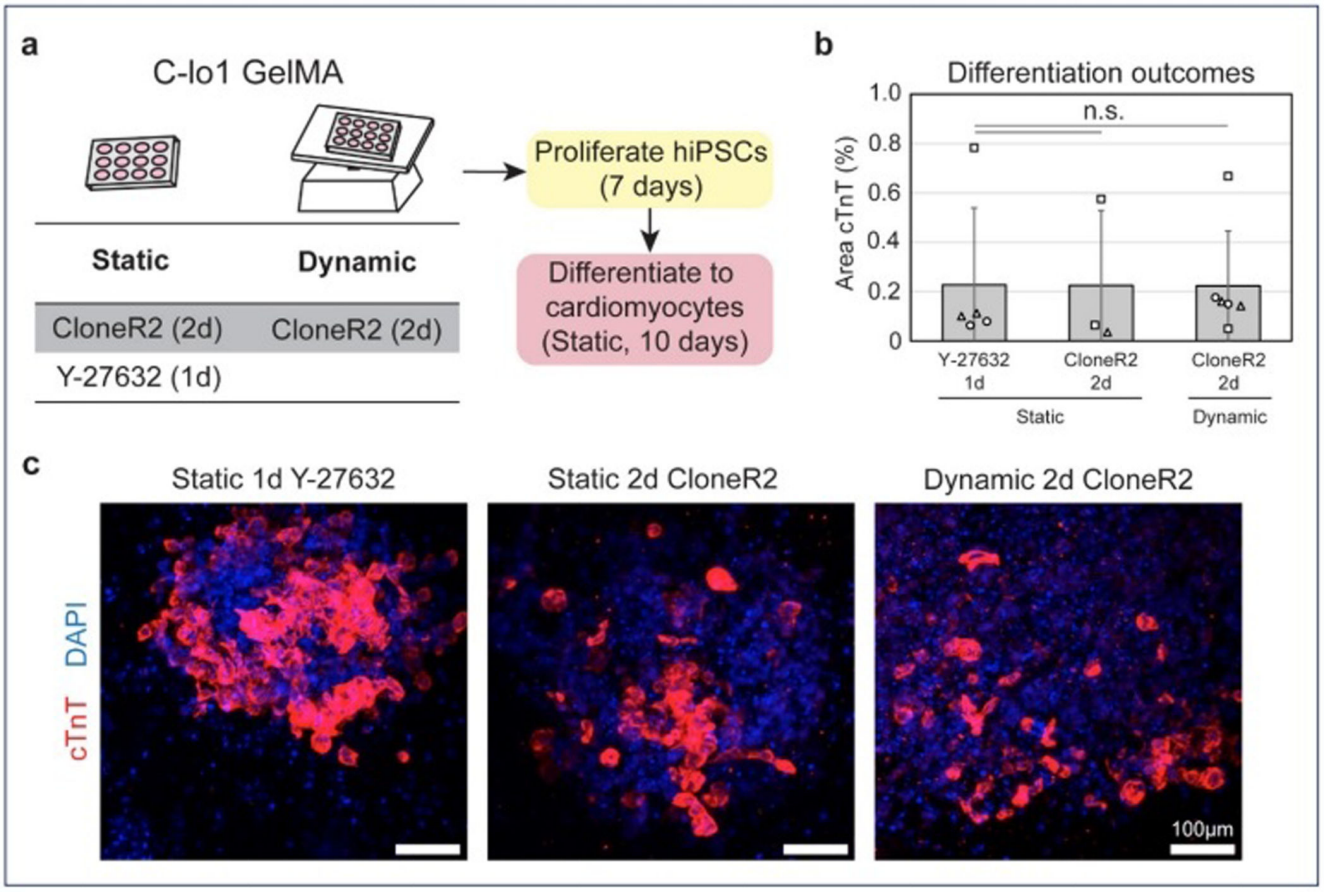
Treatment with CloneR2 does not inhibit the ability to differentiate hiPSCs from cardiomyocytes. (a) Schematic of expansion conditions tested prior to differentiation. Differentiation was initiated following 7 days of expansion. (b) The cTnT expression in differentiated tissues shows that hiPSCs in C-lo1 GelMA are able to differentiate to cardiomyocytes following treatment with static or dynamic CloneR2 culture. (c) Images of cTnT-expressing cardiomyocytes in each culture condition. (2–3 print batches per condition, batches represented by the shape of data points on bar graphs, 1–2 constructs/batch. Significance was found relative to control Y-27632 samples using Dunnett’s test. n.s. not significant.) Abbreviations: cTnT: Cardiac troponin T; GelMA: Gelatin methacryloyl; hiPSC: Human induced pluripotent stem cell.

After observing rapid hiPSC expansion with CloneR2, we next tested whether these highly proliferative cells could still differentiate into cardiomyocytes. After the 7-day proliferation phase with CloneR2 treatment, we induced differentiation of hiPSCs into cardiomyocytes in constructs made from C-lo1 GelMA (Figure 4a). After the following 10 days of cardiac differentiation, bioprinted constructs were stained for cardiac sarcomeric protein, cardiac troponin T (cTnT), to confirm differentiation outcomes. Both static and dynamic CloneR2 conditions yielded similar percentages of cTnT-positive cells compared to the Y-27632 control, indicating that CloneR2 did not negatively impact the differentiation process (Figure 4b and c). Of note, differentiation efficiency was low, likely due to the absence of additional extracellular matrix proteins known to improve differentiation outcomes, and the undifferentiated control expressed negligible cTnT (Figure S13, Supplementary File). We further tested CloneR2’s effects using a complex 3D cardiac model, the hChaMP (Figure S14, Supplementary File). Following 9 days of CloneR2-supported hiPSC expansion, cardiomyocyte differentiation was induced. Both CloneR2- and Y-27632-treated hChaMP constructs exhibited synchronous and robust beating, confirming successful formation of functional cardiac tissue (Videos S1 and S2, Supplementary File). These results demonstrate the potential of CloneR2 to support hiPSC expansion and differentiation in advanced bioprinted tissue models.

## 4. Discussion

In this study, we identified several key parameters that can impact the ability of pluripotent stem cells to expand and differentiate in 3D-bioprinted constructs. Based on these results, we provide key recommendations for future work using the ISED approach and discuss limitations that remain unsolved.

### 4.1. Primary recommendations for improving *in situ* expansion of hiPSCs in GelMA-based scaffolds

#### 4.1.1. Variability in GelMA properties emphasizes the need for reporting scaffold characterization beyond GelMA concentration

First, while GelMA is widely used for a variety of bioprinting applications, the properties of this material vary substantially among different manufacturers, despite similar DoF and gelatin starting material. Moreover, GelMA source, DoF, and concentration have a great influence on the ability of hiPSCs to expand in the constructs, which is problematic for achieving consistency in downstream differentiation applications.^40–43^ To better understand the relationship between GelMA type and hiPSC growth, we employed MSR and rheometry as metrics to describe construct material properties.^30–36^ MSR is a cheap and easy screening method to monitor differences in construct properties and can give an approximation of nutrient diffusion in a cell-laden construct.^30^ Our studies revealed that concentration, media type, and scaffold preparation technique have a more profound impact on the MSR than GelMA type. On the contrary, GelMA type greatly influenced the storage modulus, where two GelMA types with similar DoFs from different manufacturers had quite different mechanical properties. Again, due to the sensitivity of hiPSCs, this inconsistency in mechanical properties may be critical to ensuring successful ISED.^44^ These findings and a recent report by He *et al.*^51^ underscore the need to thoroughly characterize scaffold properties, especially mechanical properties, for reproducibility. Currently, GelMA concentration is the only consistently reported parameter, but as we see, the mechanical properties of 10% GelMA can vary greatly depending on manufacturer, DoF, and lot number.

#### 4.1.2. Pneumatic bioprinting and support bath characteristics influence construct material properties

Importantly, there are also several factors that can impact reproducibility in the bioprinting process itself. As bioprinted samples generated higher variability than cast samples for MSR and storage/loss moduli, even within the same ink batch, we see there is some degree of error introduced in bioprinting. While pneumatic printing offers a relatively easy-to-use biofabrication platform, it relies on a steady application of pressure with compressed gas and is easily affected by the viscosity of the bioink. Variability in viscosity among GelMA types and batches can impact the ability to generate consistent construct structures.^37–39^ We aim to circumvent this issue by adjusting pressure to a consistent drop rate of ink from the needle,^10^ but consistency of the applied pressure during printing is highly important. In many cases, setting the drop rate was not straightforward, as the drop rate could vary with the application of pressure within the same ink batch, suggesting pressure applied by the printer was not stable. This means printing outcomes are more prone to over extrusion or under extrusion, a likely cause of variability in the acellular and d0 rheometry data. As we observed a correlation between initial modulus and resulting cell growth, the lack of pneumatic stability can be a problem for achieving consistent hiPSC proliferation and differentiation. Use of a mechanical piston-driven extrusion printer may help circumvent some of these issues, as demonstrated in a recent study^39^; however, prior research of this printing modality is limited relative to pneumatic extrusion.^52^

Additionally, the use of a slurry-based support bath during printing dramatically increases porosity, as suggested by the large change in MSR between bioprinted and cast constructs. The use of a support bath is necessary for printing complex shapes with GelMA at low-viscosity concentrations that support cell growth; however, properties of the support bath, including viscosity, yield stress, and particle size, can certainly affect scaffold properties. While it has been shown that bulk hydrogels have many different properties than more porous scaffolds,^33^ we were surprised to see the stark increase in stiffness and decrease in MSR in cast samples relative to bioprinted scaffolds, presumably due to the pores created by support bath particles. Also surprising was the greater impact of saline on the swelling of bioprinted samples relative to cast, again likely due to the increased porosity in the constructs. Thus, finding a balance between print resolution and porosity with a given GelMA type is necessary for cell survival. This again emphasizes the importance of reporting details of print parameters—support bath properties in parallel with bioink and scaffold properties.

#### 4.1.3. hiPSC expansion can be quickly and non-invasively monitored via glucose consumption

When we investigated the impact of various GelMA types on hiPSC growth in bioprinted tissues, we found a strong correlation between hiPSC expansion and GelMA stiffness and DoF. We were able to easily monitor this growth with a handheld glucose meter. As cell growth can be variable (and exacerbated when switching between GelMA sources) and difficult to monitor via imaging in 3D culture (particularly with complex geometries), this tool allows us to quantify whether cells are expanding at an appropriate rate in a given GelMA type or lot. This is important for knowing the adequate time to initiate differentiation, especially for protocols that require a certain cell density for efficient differentiation. Recently reported methods for assessing cell culture in 3D scaffolds, such as Raman spectroscopy^53^ and optical coherence tomography,^54^ can provide more information on cellular behavior, and will be interesting to study in future work. However, these methods are less accessible, quantitative, and require more extensive processing to obtain results. Thus, while glucose measurements are dependent on cellular metabolism, which can be variable between cell types and associated with varied cell behaviors of a single cell type, we found this method to be reliable for hiPSC expansion. We found a high correlation between glucose consumption at early time points (even at d7 of proliferation) and colony coverage at the end of the proliferation time. This result allows for early prediction of the resulting colony coverage at the end of the proliferation period (d14).

#### 4.1.4. Substrate stiffness correlates with hiPSC expansion

We found that hiPSCs grew best in the C-lo inks, with good reproducibility between lots. However, much less expansion was seen in H-hi1 GelMA and in C-hi1 at concentrations of 5 or 10%. Thus, hiPSC growth may depend in part on DoF, with better expansion in GelMA with 50% DoF than with 95–99% DoF. Interestingly, there also appeared to be a correlation between initial G′ and final colony coverage. Few studies of 3D hiPSC culture have systematically examined the impact of substrate stiffness on hiPSC viability and expansion, despite the recent emphasis on the importance of 3D culture on biomimetic systems. Previous 2D studies seeding hPSCs on top of hydrogels have suggested an elastic modulus around 9–10 kPa was ideal for cell growth,^55,56^ though in one of these studies, hPSCs had quickly detached from the surface of the softer substrates.^55^ On the other hand, 3D studies have found high hPSC proliferation with storage modulus around 655–900 Pa.^44,57^ These relatively soft hydrogels are beneficial in that they may allow for singularized hPSCs to proliferate and grow within the matrix and may elicit similar mechanosensitive pathways used by ROCK inhibitors, as suggested in one of these studies.^44^ Here, we saw the highest expansion near a G′ of 400 ± 30 Pa, but as most hiPSCs did not survive on the highly functionalized C-hi1, which had a G′ in this range, further study is needed to evaluate the effects of other variables such as DoF, topography, or charge density^58^ on hiPSC growth. Interestingly, prior work has shown substrate mechanics can affect hPSC colony morphology,^56,59,60^ though the impact on single-cell viability has not been studied, and the effect of these changes in colony morphology on differentiation outcomes remains unclear.

During proliferation, we found a decrease in both G′ and G″ for the two C-lo inks. Matrix remodeling by stem cells has previously been shown to help maintain proliferative state, as well as pluripotency, via autocrine-induced JAK/Stat pathway.^61,62^ Interestingly, we did not observe a large change in the high DoF inks, both of which had lower hiPSC expansion. DoF and concentration have previously been shown to be inversely correlated to degradability,^34,63^ so the ability of hiPSCs to overturn the matrix in the lower DoF GelMA types and lower concentration of C-hi1 (5% relative to 10%) may have allowed for the improved expansion. This change in hydrogel mechanical properties is an important consideration for *in situ* expansion, as it may impact differentiation outcomes. Thus, identifying an optimal initial stiffness may be needed to achieve both high hiPSC expansion and high differentiation efficiency following matrix remodeling.

#### 4.1.5. Cloning supplements can improve hiPSC expansion in constructs with mechanical properties suboptimal for hiPSC proliferation

We observed some variability in final colony coverage among constructs from the same GelMA type, even from within the same ink batch, with the same bioink and cell suspension mixtures. While some of these differences may be due to the printing process, hiPSCs can also be a source of variability, with spatial differences in cell growth and variable levels of cell death upon singularization. Moreover, from monitoring glucose consumption, we found hiPSCs in some GelMA types grew fast at the beginning before plateauing around d10, apparently caused by cell death or reduced proliferation from prolonged culture, whereas other GelMA types had slow initial growth, followed by more rapid expansion later. The GelMA types with faster initial proliferation had a higher colony coverage at the d14 endpoint than those that initially grew slower. This suggests a large impact of GelMA type on early hiPSC survival, likely exacerbated upon singularization of hiPSCs.

We therefore aimed to improve survival in the first days following printing with the use of dynamic culture or media supplementation. CloneR2 has previously been used to improve single-cell survivability in cloning and transfection applications^45–47^ but has not been used for improving culture in bioprinting or culture of singularized hiPSCs in 3D scaffolds. With the use of this supplement, we found dramatic increases in glucose consumption and colony coverage in half the culture period for C-lo1 and achieved similar results with the less efficient construct, C-hi1 (5%). This demonstrates the potential to improve the culture of hiPSCs on a desired scaffold type, regardless of whether it has optimal mechanical properties. The ability to recover hiPSC expansion in C-hi1 (5%) constructs by improving single-cell viability suggests that singularized hiPSCs are more sensitive than colonies to the scaffold properties. This is supported by the Y-27632-treated C-hi1 (5%) and H-hi1 samples that grew faster at later stages, only once colonies had begun to form. While it is well-known that singularized hiPSCs are prone to cell death, it is important to note that the impact of the scaffold on hiPSC culture appears to be an important driver in cell health as well. Perhaps with further development of methods to improve single-cell hiPSC viability, the impact of the scaffold on hiPSC culture will be less notable and variability will be further reduced. This will allow for greater options in scaffold mechanical properties that can be used for ISED applications.

### 4.2. Major limitations

In addition to the sources of variability described above, there are also many factors that create variability in the GelMA source, which are much harder for the user to control, and that exist concerning whether GelMA is made in-house or purchased commercially. These factors include the biophysical properties of the gelatin that affect macroscale properties,^64^ batch-to-batch variabilities of the gelatin reactive groups available for functionalization,^65^ and difficulties replicating the DoF. Moreover, the choice of gelatin source (animal source, type A vs type B, and bloom strength) used for producing GelMA can greatly impact scaffold properties.^40–43^ Even quantification of DoF can give varying outputs depending on the assay used, which is problematic for studying the impact of DoF on cell viability. Regardless of method used for quantification (colorimetric, ^1^H NMR, *etc.*), the choice of standard is crucial for accurate results, requiring the use of the original gelatin material to account for the inherent variability of gelatin.^65^ This can make it difficult to compare products from various vendors, which either do not list the DoF or give a large range in their product details. Additionally, assays to detect functionalization with both methacrylate and methacrylamide groups are necessary for full characterization of the material.^63,66^ Another limitation includes the shelf-life of GelMA, which is typically around 6–12 months for commercial products, making use of the same lot of GelMA for a given application difficult. Further, products are usually sold in discrete quantities, requiring solubilization of a large batch of ink at once, or aliquoting of the lyophilized product, which increases exposure risk to humidity and thereby impacts the shelf life.

Overall, these features emphasize the need for a consistent product, as well as more thorough characterization of GelMA scaffolds reported in the literature for improved reproducibility. Fortunately, some groups have reported improved methods for reducing batch-to-batch variability in GelMA production with the promise to overcome many of these limitations.^63,67^ This is accomplished via a one-pot synthesis that raises the pH greatly above the isoelectric point of the gelatin for a complete reaction, with the addition of a buffer to maintain the pH throughout the reaction. This method has been demonstrated to lead to consistent DoF, as well as reproducible mechanical properties and cell viability, across 5 batches.^63^

Another relevant consideration on scaffold variability is current bioprinter technologies. Holding bioinks constant, a recent study examining the reproducibility of extrusion printing among 12 research groups demonstrated the immense variability that can occur due to user or bioprinter.^39^ Researchers from each group were trained; labware, STL files, and bioink were identical; and analysis of resulting constructs was automated. Yet, inter- and intra-laboratory error in print fidelity existed, leaving room for future improvements in bioprinter development. For instance, use of flow meters on pneumatic printers may aid in controlling extrusion.^68^

In addition to the inherent limitations of GelMA and bioprinting, limitations remain in hiPSC culture as well. The results found here demonstrate that the effect of the scaffold on cell growth may be less pronounced with reduced death upon singularization. Thus, printing with colonies rather than singular hiPSCs may be beneficial. Generating consistent colony sizes would remain a challenge, although use of dextran sulfate has been shown to improve homogeneity of hPSC aggregate size,^69^ and recent advances in bioreactor technology would allow for larger scale-up of these aggregates for printing.^70^ For extrusion bioprinting, this would require a larger nozzle diameter, which will reduce print resolutions, and an increase in bioink viscosity to reduce colony dissociation.^11^ Alternatively, printing could be performed via photolithography, though this is still an underdeveloped technology, with limitations including long printing times that may impact cell viability and challenges in multi-material printing.^71,72^ However, as hiPSC applications develop, we will identify more methods of reducing variability in culture, both within a given cell line, and among different lines, especially key to effectively culturing patient-derived lines for translational applications.

## 5. Conclusion

In summary, our study evaluates several factors that can influence the growth of hiPSC in 3D environment, including scaffold mechanical properties, variability among GelMA samples, printing parameters, and the viability of singularized hiPSCs. We successfully employed a handheld glucose meter for rapid, quantitative, and non-invasive monitoring of cell growth. Our results indicate a strong dependence of hiPSC survival and expansion on the source of GelMA, with significant correlations to DoF and construct mechanical properties. Thus, we emphasize the importance of characterizing and reporting scaffold and printing parameters to enhance reproducibility across research groups. Importantly, we are able to recover some of the inhibitory effects of construct mechanical properties on hiPSC growth via the use of a cloning supplement that reduces the initial cell death of singularized cells. Overall, these insights are valuable for researchers developing hiPSC-laden 3D tissues for various *in situ* expansion and differentiation applications.

## Supplementary Material

Supplementary Materials

Video S1

Video S2

## Figures and Tables

**Table 1.** GelMA sources and lot numbers.

Label	Source	Cat#	DoF (%)	Lot
**C-lo1**	Advanced Biomatrix	VL3500000502	50	9385
**C-lo2**	Advanced Biomatrix	VL3500000502	50	9521
**C-lo3**	Advanced Biomatrix	VL3500000502	50	9237
**C-hi1**	Advanced Biomatrix	5208	95	9272
**C-hi2**	Advanced Biomatrix	5208	95	8622
**H-hi1**	University of Minnesota Bioprinting Facility	G99	99	9
**H-hi2**	University of Minnesota Bioprinting Facility	G99	99	10

Note: Materials were used at 10% concentration, unless otherwise noted. Three GelMA types were sourced from either a commercial supplier (“C”) or an in-house facility (“H”), with two different DoFs [(high (hi) and low (lo))]. Each GelMA type included 2–3 lots (“1,” “2,” or “3”) for comparison.

Abbreviation: DoF: Degree of functionalization.

## Data Availability

All data that support the findings of this study are included within the article (and the supplementary files).
