# Supplementary material for: Toward robust and reproducible pluripotent stem cell expansion in bioprinted GelMA constructs
Source: Int J Bioprint. Author manuscript; Available in PMC 2025 May 5. (PMC12052315; doi:10.36922/ijb.4633)
Supplement: Supplementary Materials [file NIHMS2048393-supplement-Supplementary_Materials.pdf]

International
Journal of Bioprinting

RESEARCH ARTICLE

Toward robust and reproducible pluripotent stem cell expansion in bioprinted GelMA constructs

Supplementary File

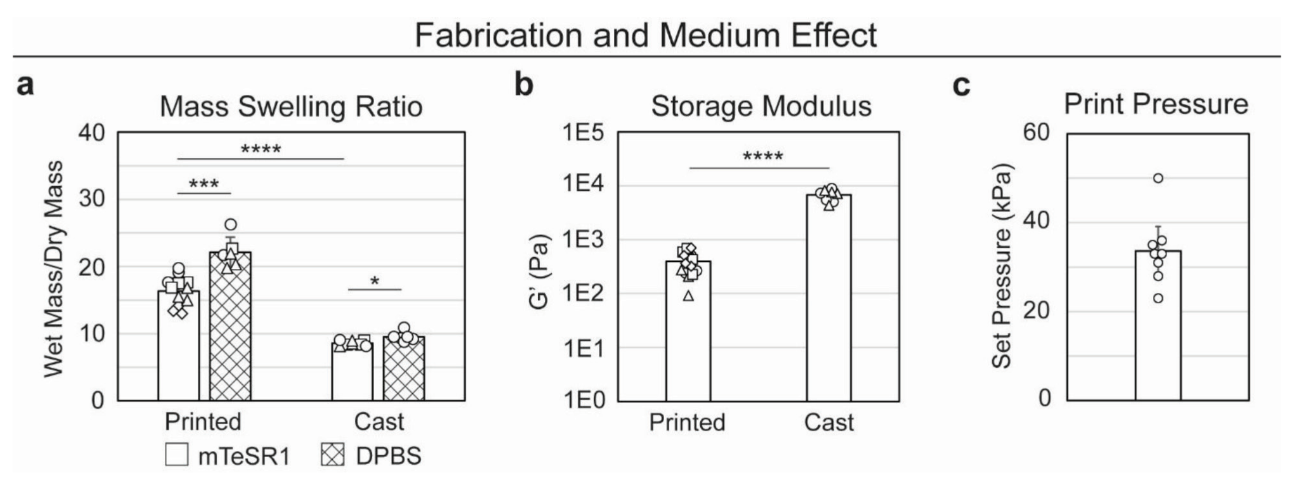

Figure S1. GelMA fabrication method and hydration medium affect scaffold mass swelling and mechanical properties. (a–b) The culture of constructs (C-hi1 GelMA) in DPBS rather than mTeSR1 causes a large increase in MSR (a). Casting GelMA rather than bioprinting causes a large drop in MSR (a) and a significant increase in G′ (b), while also reducing variability. (c) Set printing pressures (C-hi1 GelMA) exhibit variability, ranging from 23 to 50 kPa. (2–4 print batches per GelMA type, batches represented by different shapes of data points on bar graphs, 2–3 constructs/batch for MSR, 3–4 constructs/batch for rheometry. *$p \leq 0.05$, ***$p \leq 0.001$, ****$p \leq 0.0001$.) Abbreviations: GelMA: Gelatin methacryloyl; MSR: Mass swelling ratio.

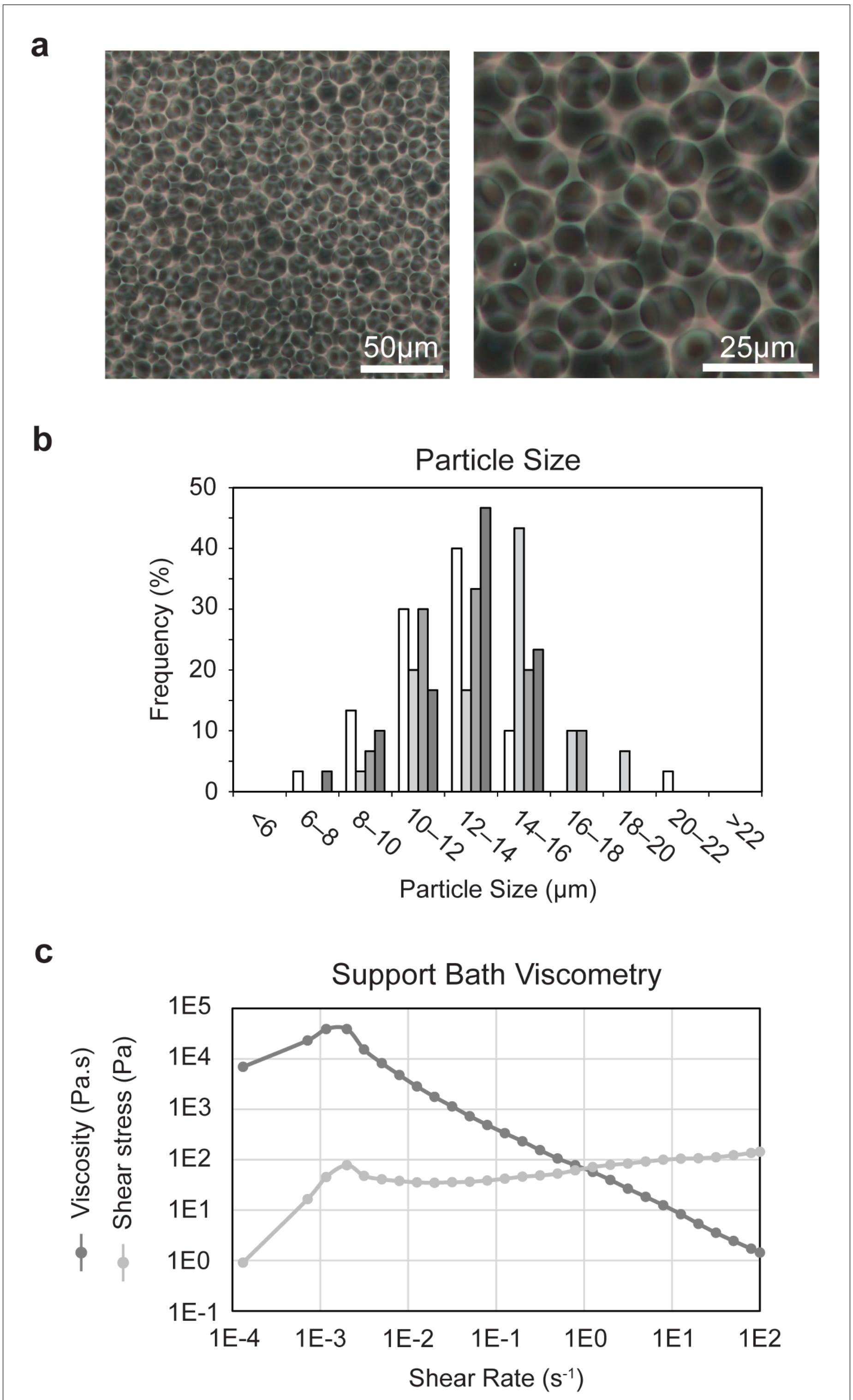

Figure S2. Support bath characterization. (a) Image of microparticles in the support bath, non-packed, prior to centrifugation. (b) Distribution of particle sizes among 4 print batches, with an average diameter of 12.98 ± 2.26 µm (30 particles quantified per batch). (c) Viscometry measurement of the support bath gives a yield stress of 75.6 ± 34.1 Pa (determined from 3 batches, representative plot shown).

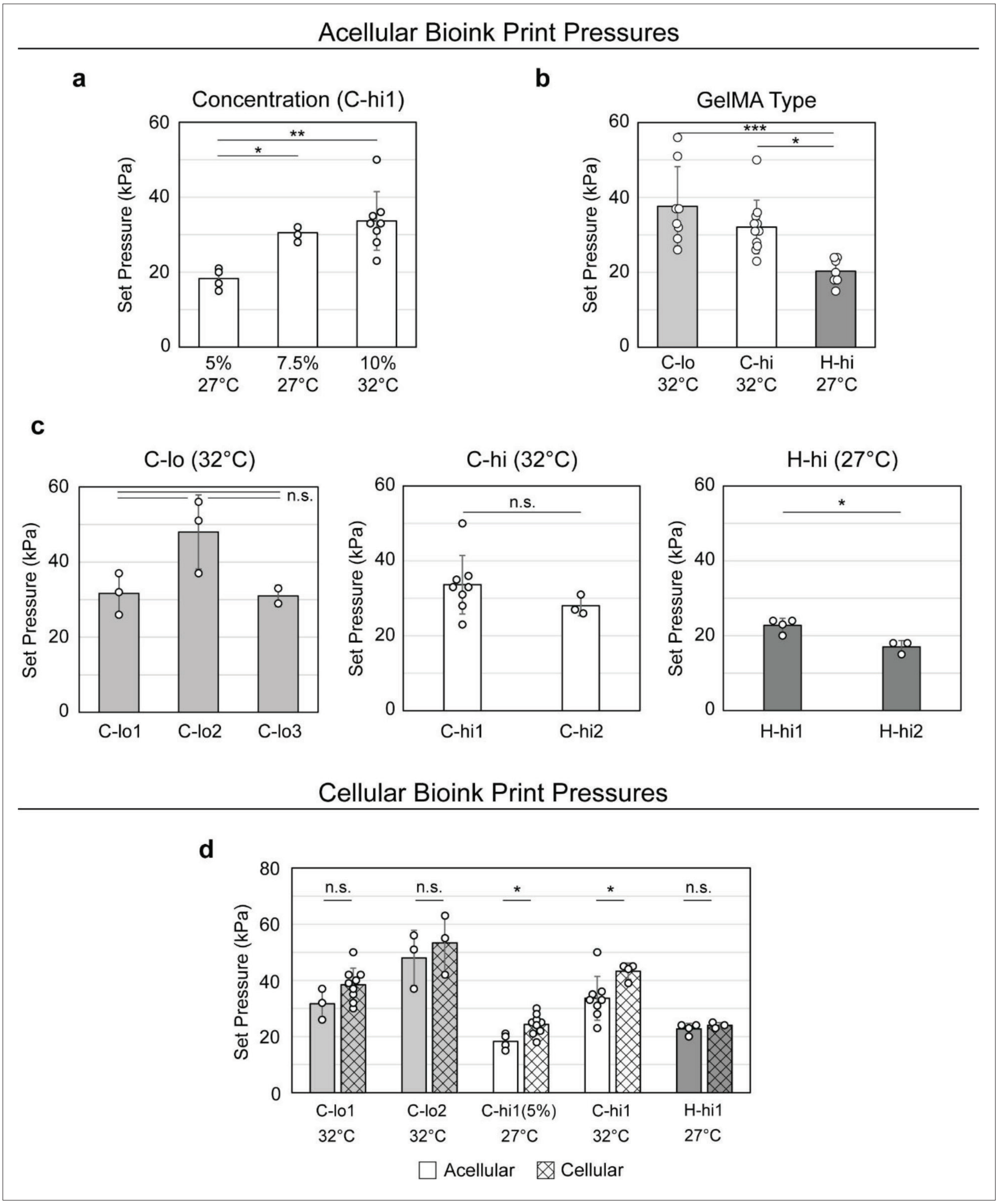

Figure S3. Print pressure and temperature vary by GelMA type and concentration. Required printing pressures at the given temperatures for acellular (a–c) and cell-laden bioinks (d). (a) Print pressure increased with GelMA concentration, despite the increase in temperature used at 10% concentration. (b) Inks from source C required higher printing pressures and temperatures than those from source H. (c) Little variability existed within lots for C-lo and C-hi, but H-hi inks were statistically different. (d) A slight increase was required when printing with cells for each GelMA type. While only 2 of 5 GelMA types had non-significant increases via *t*-tests, two-way ANOVA with all data suggests the presence of cells to be a significant factor in print pressure (p = 0.0026). (2–8 print batches per GelMA type. *$p \le 0.05$, **$p \le 0.01$, ***$p \le 0.001$, n.s. not significant.) Abbreviations: ANOVA: Analysis of variance; GelMA: Gelatin methacryloyl.

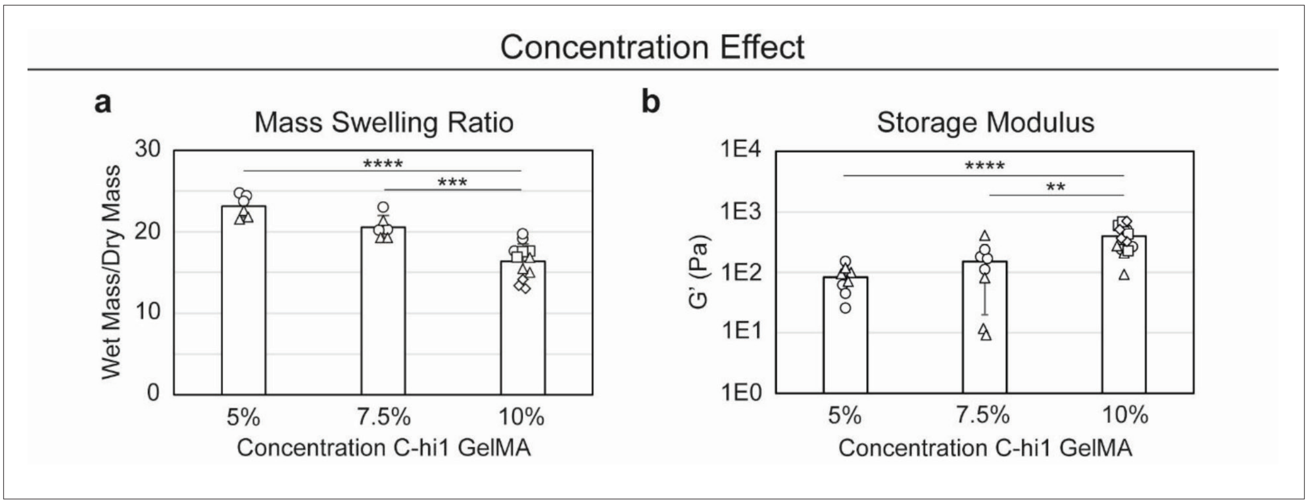

Figure S4. The concentration of GelMA affects scaffold properties. Scaffold properties were found to change with GelMA concentration, where concentration was inversely proportional to MSR (a) and directly proportional to G′ (b). (2–4 print batches per concentration, batches represented by shape of data points on bar graphs, 2–3 constructs/batch for MSR, 3–4 constructs/batch for rheometry. $^{**}p \le 0.01$, $^{***}p \le 0.001$, $^{****}p \le 0.0001$.) Abbreviations: GelMA: Gelatin methacryloyl; MSR: Mass swelling ratio.

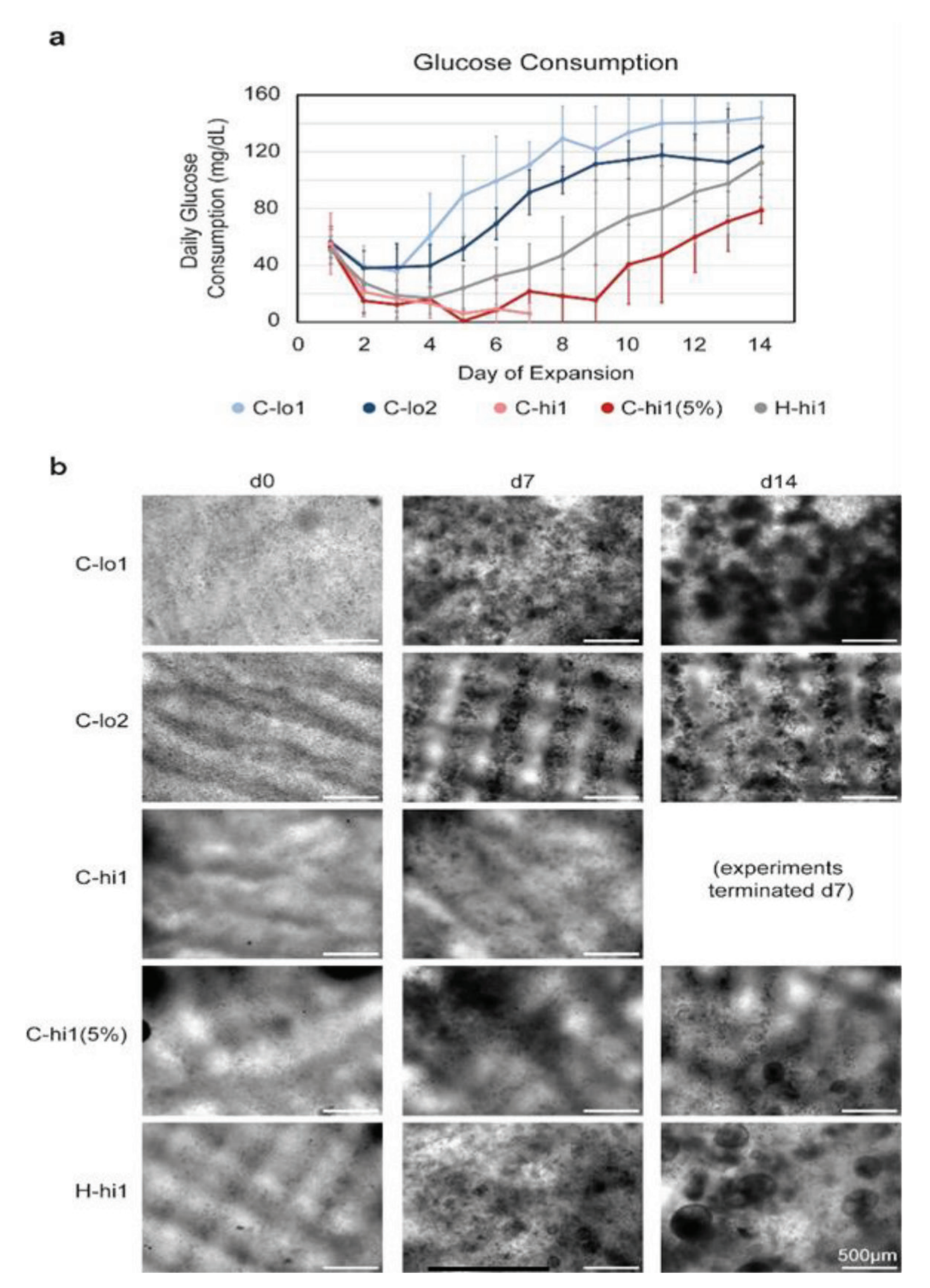

Figure S5. hiPSC expansion varies across GelMA types. (a) Glucose was monitored daily during the 14-day proliferation for each ink type to quantitatively approximate *in situ* cell growth. (n = 2–3 measurements per point, exhausted media of similar conditions were pooled within each replicate prior to measurement). (b) Brightfield images showing hiPSC expansion. Grid-like patterns reflect print lines from the rectilinear infill; print lines generally become less noticeable as cells proliferate and potentially remodel the constructs. Abbreviations: GelMA: Gelatin methacryloyl; hiPSC: Human induced pluripotent stem cell.

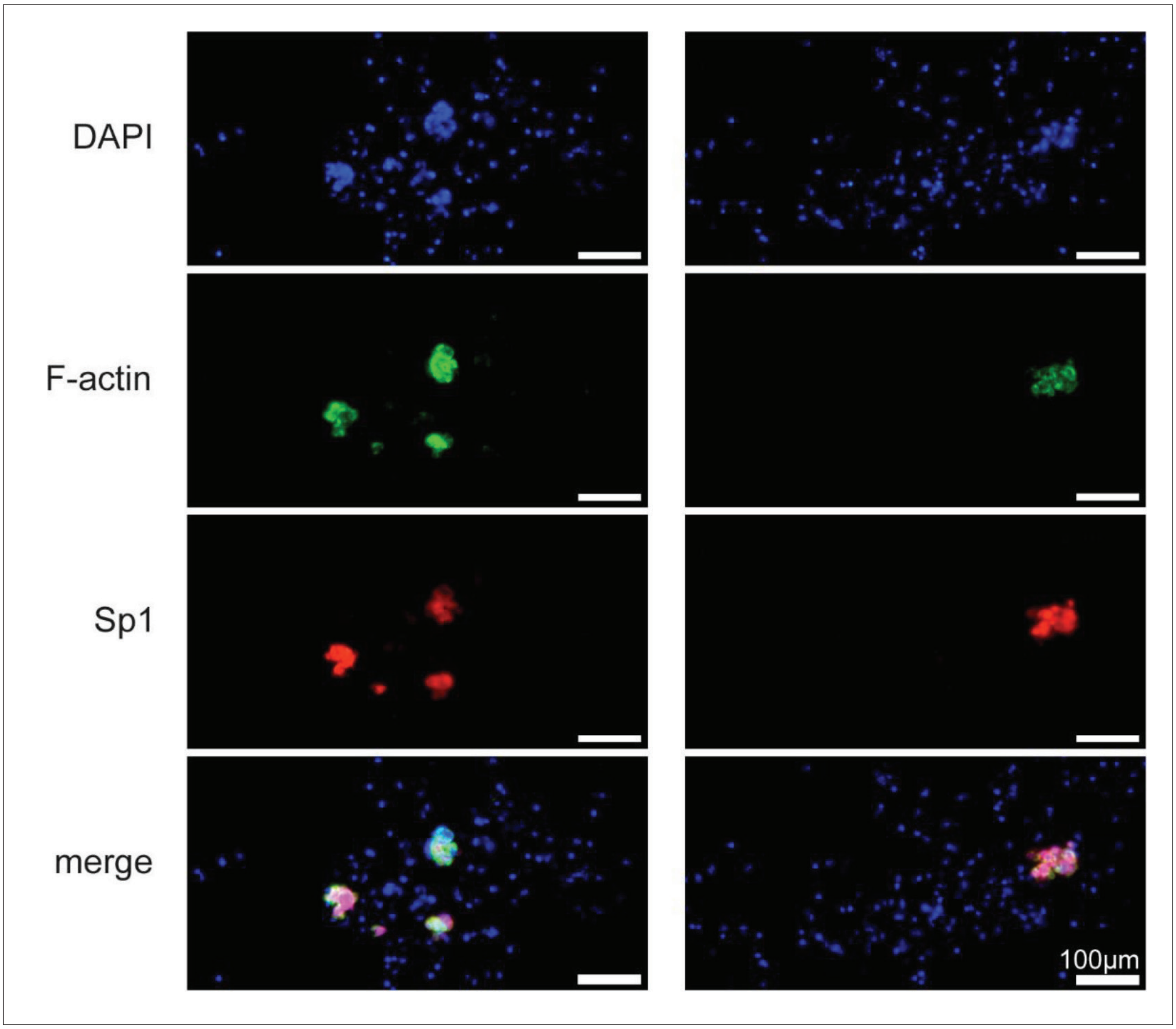

Figure S6. F-actin and Sp1 are expressed in colonies only. F-actin and Sp1 are expressed in cells in colonies but not in singularized cells at the end of the proliferation period (d14), confirming that only cells in colonies are viable.

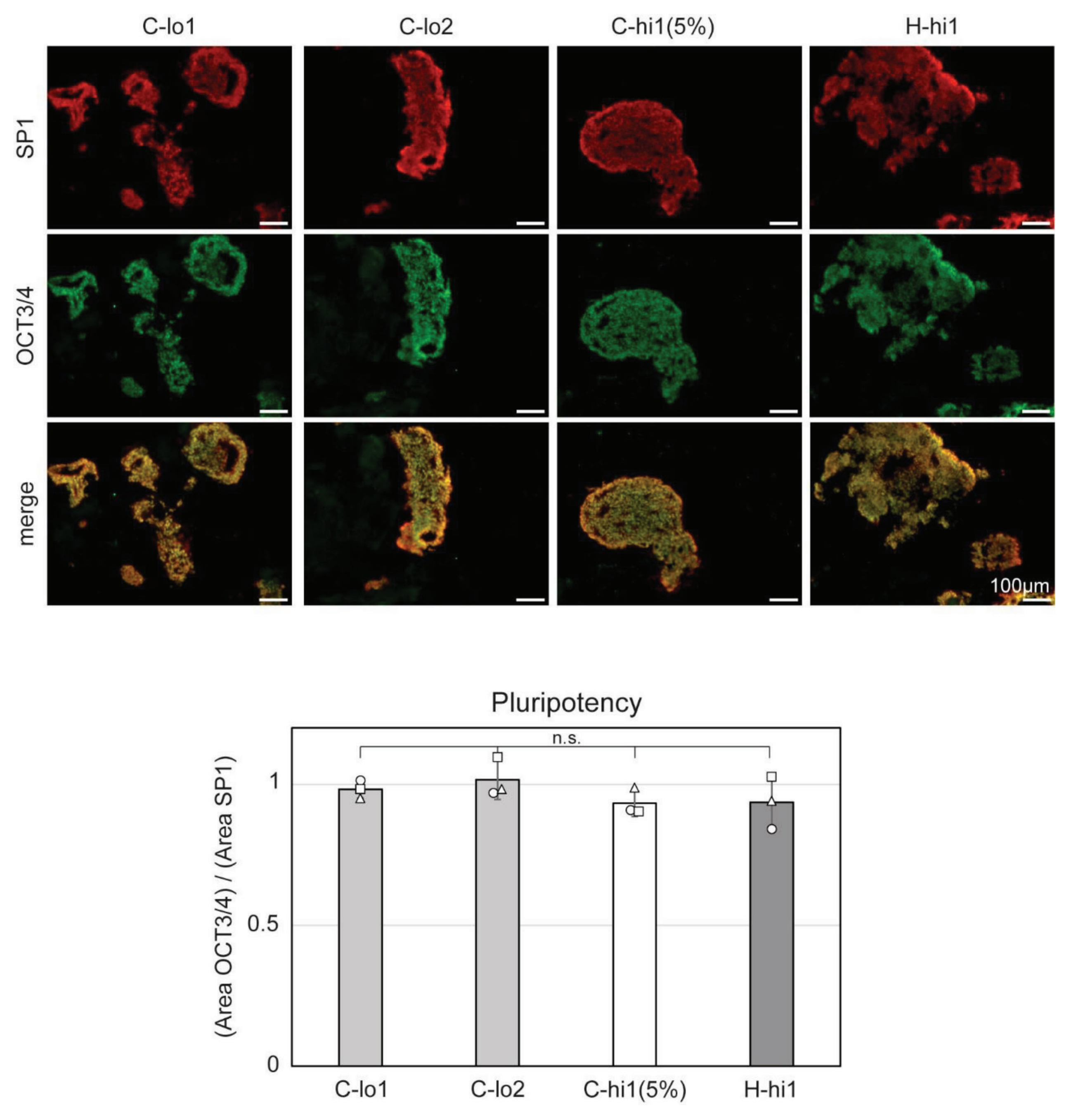

Figure S7. hiPSC remains pluripotent in each GelMA type. Levels of pluripotency marker OCT3/4 remain high in all colonies of each GelMA type. For quantification of live cells only, OCT3/4 expression was normalized to the expression of ubiquitous protein SP1. (3 print batches per GelMA type, batches represented by the shape of data points on bar graphs. n.s. not significant.)

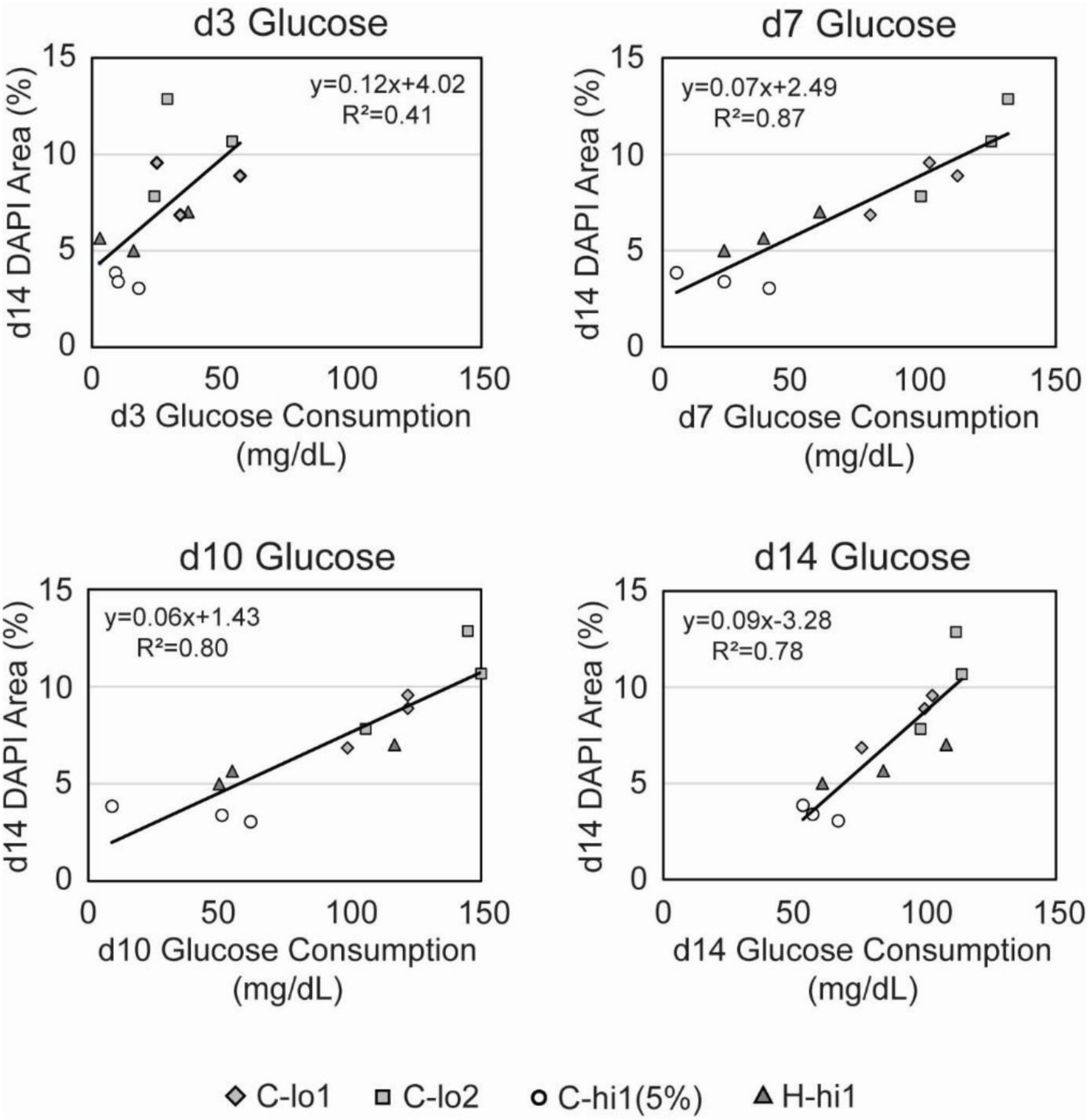

Figure S8. Glucose consumption correlates to final colony coverage. Glucose consumption at d3 appears to be too early to be indicative of colony coverage, which is more accurate at d14 of expansion, although d7, d10, and d14 are already highly correlative to the final coverage.

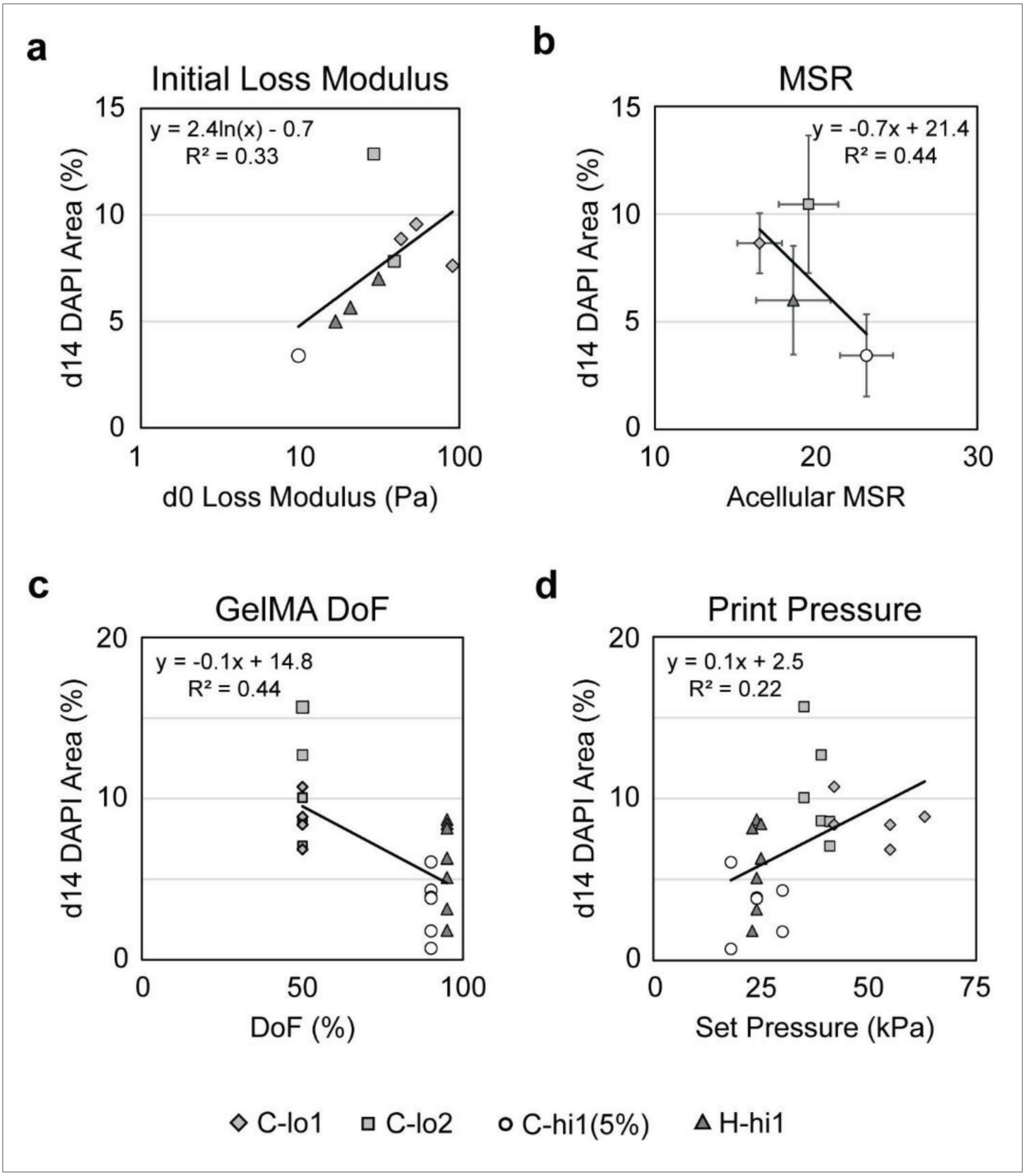

Figure S9. GelMA scaffold and ink properties impact on hiPSC expansion. Pooling data across the four GelMA types shows that G″ (a), like G′, may affect expansion. GelMA properties like MSR (b, using averages of acellular results) and DoF (c) may also be important variables. Set print pressure (d) appears to have less influence on hiPSC expansion.Abbreviations: DoF: Degree of functionalization; GelMA: Gelatin methacryloyl; hiPSC: Human induced pluripotent stem cell.

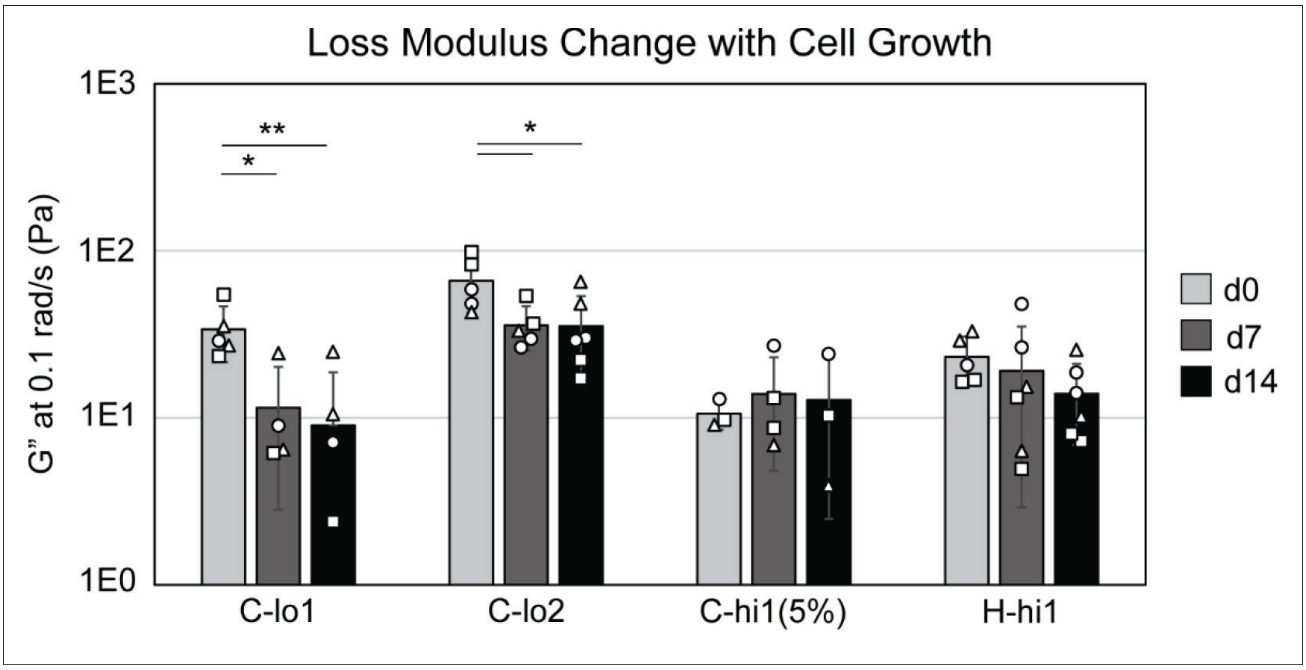

Figure S10. Loss modulus changes with cell growth in C-lo GelMA. Like G′, G″ was found to decrease with expansion for C-lo1 and C-lo2 GelMA types, but with little change in C-hi1 (5%) and H-hi1 (3 print batches per GelMA type, batches represented by shape of data points on bar graphs, 1–3 constructs/batch. $^*p \leq 0.05$, $^{**}p \leq 0.01$.) Abbreviation: GelMA: Gelatin methacryloyl.

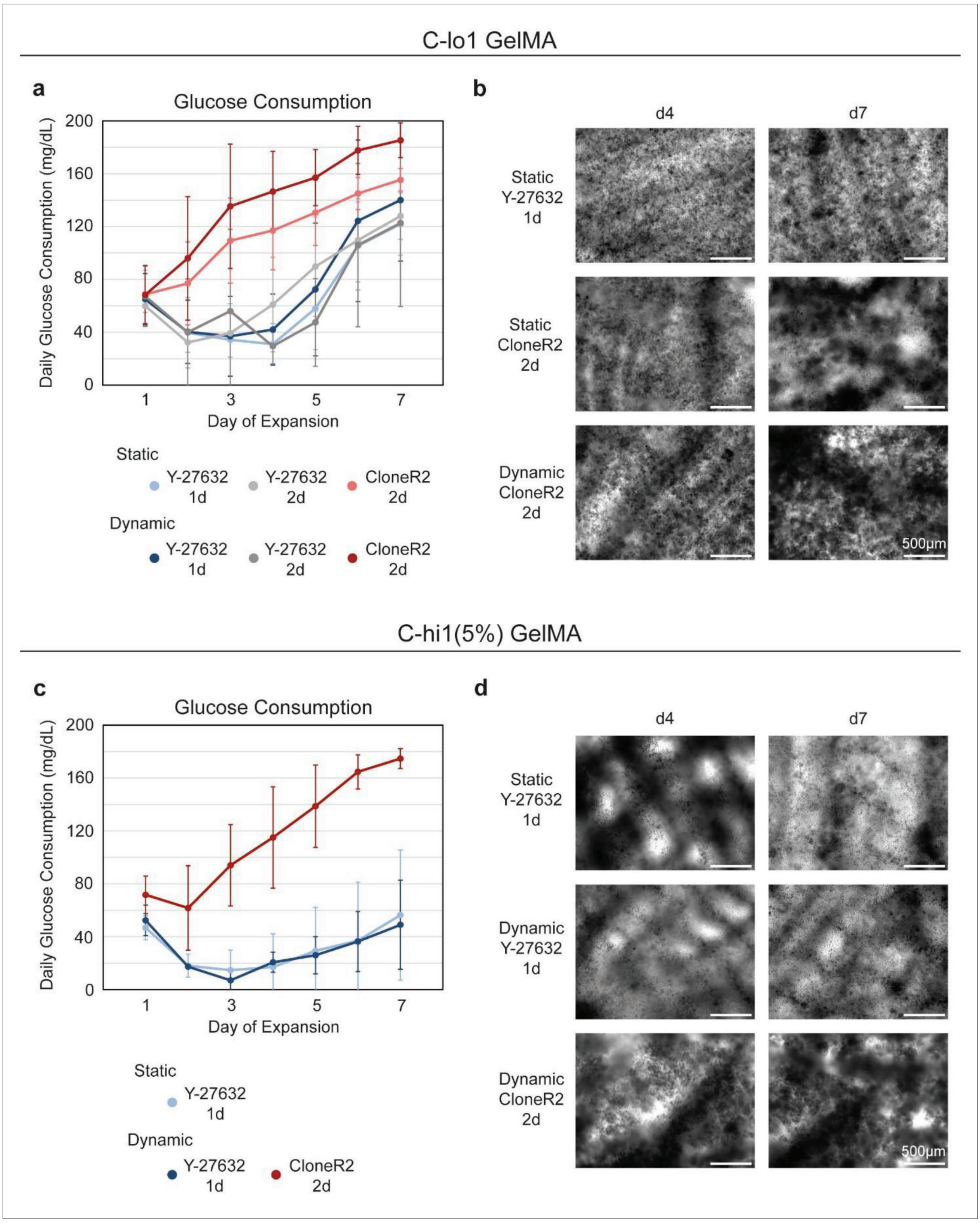

Figure S11. The use of cloning supplements accelerates hiPSC expansion despite the GelMA type. (a) Glucose was monitored daily during the 7-day proliferation for C-lo1 to quantitatively approximate *in situ* cell growth under each culture condition. CloneR2-treated constructs caused accelerated glucose consumption. (b) Images showing cell growth and colony formation with culture under control and CloneR2 treatment conditions in C-lo1. (c) Glucose consumption during the 7-day proliferation in C-hi1 (5%) upon treatment with different culture conditions. CloneR2 caused high glucose consumption, with low variability. (d) Cell growth and colony formation are only found with the use of dynamic CloneR2 in C-hi1 (5%) GelMA. (For (a) and (c), n = 2–3 measurements per point, exhausted media of similar conditions were pooled within each replicate prior to measurement. For (b) and (d), grid-like patterns reflect print lines from the rectilinear infill; print lines generally become less noticeable as cells proliferate and potentially remodel the constructs.) Abbreviations: GelMA: Gelatin methacryloyl; hiPSC: Human induced pluripotent stem cell.

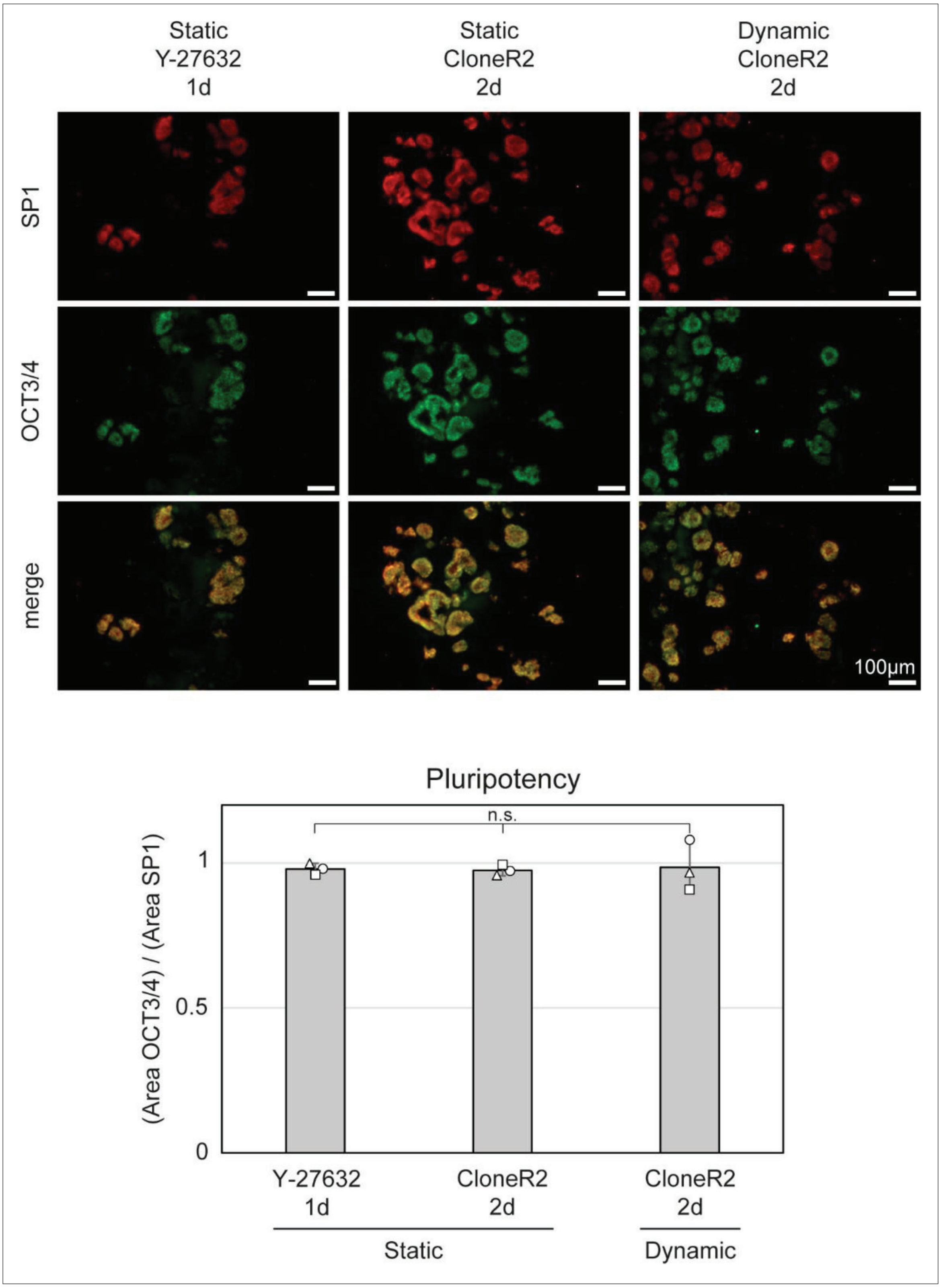

Figure S12. hiPSCs remain pluripotent after culture with CloneR2. Levels of pluripotency marker OCT3/4 remain high in all colonies following 2-day treatment with CloneR2. For quantification of live cells only, OCT3/4 expression was normalized to the expression of ubiquitous protein SP1. (3 print batches per GelMA type, batches represented by the shape of data points on bar graphs. n.s. not significant.)

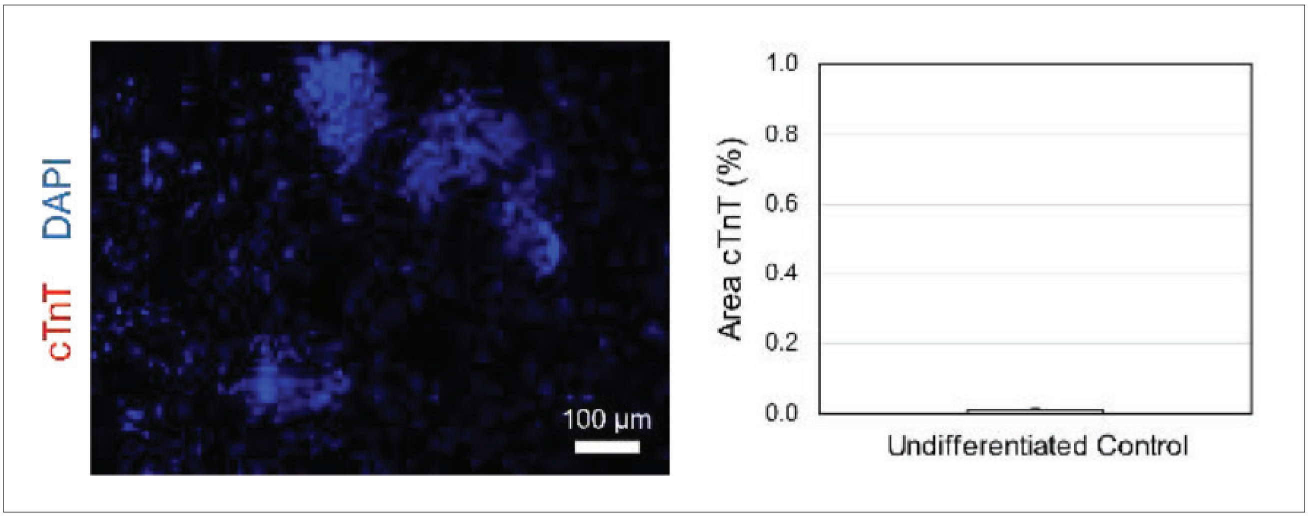

Figure S13. Spontaneous cardiac differentiation from hiPSCs does not happen without differentiation media and CloneR2 treatment. Cardiac troponin T (cTnT) expression is minimal (0.011 ± 0.005 %) in the control without differentiation media and CloneR2. Bioprinted constructs were stained for cTnT and with DAPI (3 constructs, 5 images per construct were used for quantification).

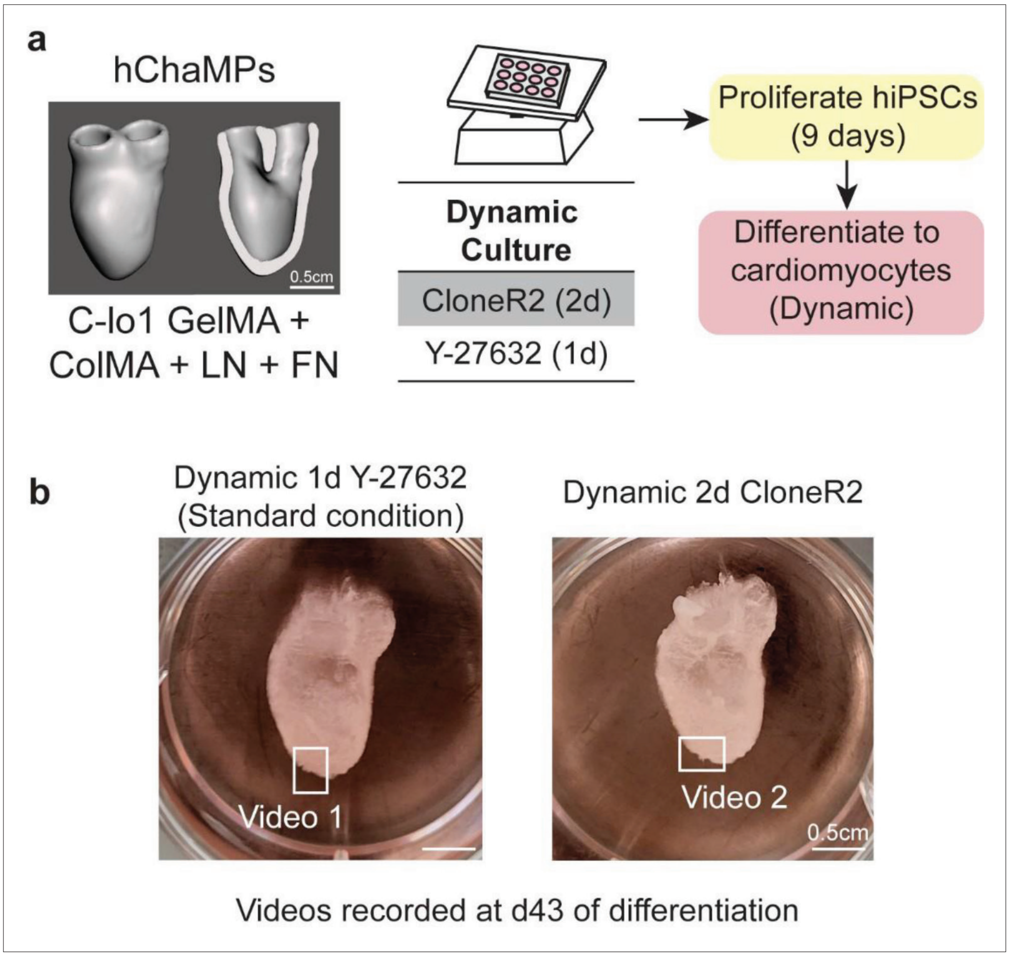

Figure S14. Differentiation of hiPSC-laden hChaMP to cardiomyocytes following hiPSC expansion with dynamic CloneR2 treatment. (a) Schematic of hChaMP design and bioink (containing GelMA with ColMA, LN, and FN; left) and treatment regimen (right). (b) Image of hChaMPs following differentiation; white rectangles designate regions represented in Videos S1 and S2. Abbreviations: ColMA: Methacrylated collagen; FN: Fibronectin; GelMA: Gelatin methacryloyl; hChaMP: Human chambered muscle pump; hiPSC: Human induced pluripotent stem cell; LN: Laminin.

Supplementary Videos

Video S1. Y-27632-treated hChaMP shows macroscale contraction at d43 of differentiation. The recorded region of the tissue is denoted in **Figure S14b**.
Video S2. CloneR2-treated hChaMP shows macroscale contraction at d43 of differentiation. The recorded region of the tissue is denoted in **Figure S14b**.
